# Dynamics of Cell Fate Decisions during Chemically Induced Multi‐Lineage Trans‐Differentiation at Single‐Cell Level

**DOI:** 10.1002/advs.202409642

**Published:** 2025-03-07

**Authors:** Weigao E, Lijiang Fei, Jingjing Wang, Xinru Wang, Renying Wang, Xueyi Wang, Peijing Zhang, Jianhui Chen, Junqing Wu, Mengmeng Jiang, Daosheng Huang, Danmei Jia, Guoji Guo, Xiaoping Han

**Affiliations:** ^1^ Bone Marrow Transplantation Center of the First Affiliated Hospital, and Center for Stem Cell and Regenerative Medicine Zhejiang University School of Medicine Hangzhou Zhejiang 310000 China; ^2^ Liangzhu Laboratory Zhejiang University 1369 West Wenyi Road Hangzhou 311121 China; ^3^ School of Medicine Tsinghua University Beijing 100191 China; ^4^ Zhejiang Key Laboratory of Multi‐omics Precision Diagnosis and Treatment of Liver Diseases Hangzhou Zhejiang 310000 China

**Keywords:** MEF, Single cell, Stem cell, transdifferentiation

## Abstract

Cell trans‐differentiation offers a powerful means to manipulate cell identities. By exposing cells to a combination of small molecules (SMs), cell trans‐differentiation can be induced in a simple and cost‐effective manner. However, a comprehensive atlas detailing chemical‐induced cell trans‐differentiation across multiple cell fates has yet to be established. In this study, the underlying mechanisms of trans‐differentiation is investigated and constructed an in‐depth single‐cell atlas of this process. The time‐course trajectory is demonstrated for trans‐differentiation of mouse embryonic fibroblasts (MEFs) into multiple cell lineages including epithelial, neural, extraembryonic endoderm like (XEN‐like) cells, and endothelial cells, when induced by SMs cocktail 6TCF (E616452, tranylcypromine, CHIR99021, and forskolin). These trans‐differentiated cells closely resemble various somatic cell types in the fetus. It is found that trans‐differentiation is marked by dynamic shifts in entropy and the cell cycle during cell fate transitions. A common intermediate feature is revealed characterized by high ribosomal gene expression. This study combines high‐resolution landscape with comparative analyses of trans‐differentiation dynamics, providing new insights into the complex mechanisms driving cell fate determination in vitro. Future study shall explore the applicability of the model in human cell trans‐differentiation.

## Graphical abstract

A critical aspect of chromosomal remodeling in small molecule‐induced multilineage trans‐differentiation(iMT) is elucidated. A dynamic atlas spanning over 35 days profiles terminal trans‐differentiated cells, which reverted to a more youthful state. This study reveals parallels between iMT and other cell fate determination processes, emphasizing an intermediate state characterized by heightened potential and increased cell cycle activity.

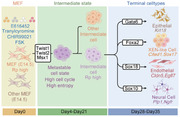

## 1 Introduction

Trans‐differentiation, a process to allow for direct cell lineage conversion^[^ ^1^ ^]^ can be achieved through forced expression of key transcription factors (TFs).^[^ ^2^, ^3^ ^]^ Alternatively, SMs can be used to reprogram somatic cells, offering a genome integration free method for the development of regenerative therapies.^[^ ^4^, ^5^, ^6^, ^7^ ^]^ In our previous study,^[^ ^8^ ^]^ we found that MEFs will be induced to trans‐differentiate into a wide range of somatic lineages simultaneously by treatment with a combination of 6TCF chemical cocktail, during which no expression of the stem cell marker Oct4 was detected. We termed this phenomenon as induced multilineage trans‐differentiation (iMT). iMT can be modified for directional lineage trans‐differentiation through different chemical combinations. Single cell qPCR analysis uncovered a novel priming state that enables transition from fibroblast cells to diverse somatic lineages. Despite its multiple trans‐differentiation potential, the precise cellular composition and regulatory mechanisms governing the dynamics of cell fate transition remain to be elucidated. Further research is necessary for fully understanding and harnessing the potential of this technique in the fields of cell biology and regenerative medicine.

In the last decade, the power of single‐cell RNA sequencing (scRNA‐seq) technologies has been leveraged to better investigate the trans‐differentiation process.^[^ ^9^ ^]^ Several research groups have employed scRNA‐seq to reconstruct cellular trajectories from fibroblasts to specific cell types, such as neurons^[^ ^10^ ^]^ and cardiomyocytes,^[^ ^11^ ^]^ via pioneer factors. Moreover, in a recent study, scRNA‐seq was used to map cells that were exposed to chemical compounds to induce the conversion of somatic cells to pluripotent stem cells.^[^ ^12^ ^]^ However, these studies primarily focused on delineating a single lineage reprogramming trajectory. The molecular principles underlying chemical induced trans‐differentiation into multiple lineages need to be further deciphered.

The mechanism of cell fate determination is a fundamental issue in biology. Natural processes such as development and regeneration represent natural cell fate determination, while trans‐differentiation and de‐differentiation involve artificial manipulation of cell fate. Despite their differences, these processes may share common pathways. Exploring the mechanisms of trans‐differentiation in detail can not only advance our understanding of development and regeneration but also reveal the intricate genome encoding of cell fate status.

In this study, we employed RNA‐seq, ATAC‐seq, and CUT&Tag technologies to investigate gene expression and epigenetic changes during the iMT process. By leveraging high‐throughput sequencing, we aimed to gain deeper insights into the underlying molecular mechanisms, complementing the phenotypic observations. Our results demonstrate the emergence of multiple terminal cell types, with chromosomal remodeling playing a crucial role in this process. Additionally, we applied scRNA‐seq to map over 300000 individual cells from nine MEF cell lines across ten time points, spanning a 35‐day period. This extensive dataset revealed a diverse array of trans‐differentiation trajectory, including epithelial, endothelial, neural, and XEN‐like cells. These cell states exhibited gene expression and TF profiles closely resembling those of in vivo counterparts, with some cells transitioning into a more rejuvenated state. Our analysis further revealed a common intermediate feature characterized by high ribosomal gene expression. By integrating our results with data from other processes of cell fate determination^[^ ^3^, ^12^, ^13^, ^14^, ^15^ ^]^ such as embryonic development, regeneration, and de‐differentiation—we identified a shared “initiation” state marked by high entropy and elevated cell cycle activity. This common state serves as a key precursor to the various reprogramming trajectories, shedding light on the dynamic processes governing cell fate determination. Our study offers new perspectives on the shared mechanisms that guide cell fate transitions in different biological contexts.

## 2 Results

### 2.1 A Multi‐Omics Roadmap for the iMT Process

**Figure 1:**
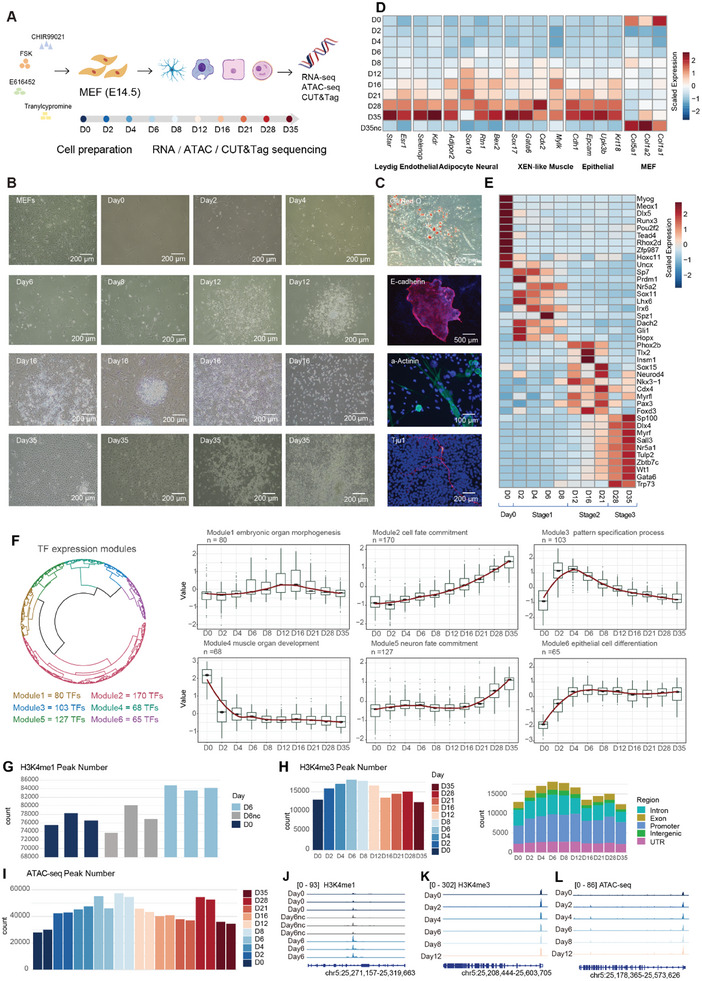
Multi‐Omics Reveals Dramatic Alterations in Gene Expression and Epigenetic Patterns during iMT Process. A) Overview of the experimental and bioinformatics workflow. B) Cellular morphological changes during the iMT process. C) Molecular characterization of different iMT cell types by Oil red O staining (adipocyte) or immunostaining of E‐cadherin (epithelial),α‐Actinin (cardiac myocyte), and Tuj1 (neuron). DAPI staining is shown in blue. D) A heatmap demonstrating the gene expression of markers for different terminal cell types (rows) across ten time points (columns) in iMT process. “D0” to “D35” represent cells cultured in iMT medium for 0 to 35 days. “D35nc” represents cells cultured in MEF medium for 35 days as a negative control. E) A heatmap demonstrating the TF expression (columns) of 4 stages (rows) during iMT process. F) A circular dendrogram illustrating clustering of TF expression pattern modules over time in RNA‐seq data (left). The boxplots present the average expression of TFs (columns) from each cluster at each time point (rows) based on the clustering results on the right, along with the most significantly enriched GO terms for the corresponding TF module (right). The red line represents the fitting curve. G) A barchat showing the numbers of H3K4me1 peaks (column) of cells in three conditions (row), 3 replicates for each condition. “D0” and “D6” represent cells cultured in iMT medium for 0 and 6 days. “D6nc” represents cells cultured in MEF medium for 6 days. H) Barcharts demonstrating the number (left) and the genomic location (right) of H3K4me3 peaks across ten time points. I) A barchat showing the numbers of ATAC‐seq peaks (column) across ten time points (row), 2 replicates for each time point. J) Normalized sequencing tracks of H3K4me1 at Kmt2c regions across three conditions, 3 replicates for each condition. “Day0” and “Day6” represent cells cultured in iMT medium for 0 and 6 days. “Day6nc” represents cells cultured in MEF medium for 6 days. K,L) Normalized sequencing tracks of H3K4me3 (K) and openness (L) at Kmt2c regions across different time points.

In our previous study, we demonstrated that MEFs can be simultaneously induced into multiple somatic cell lineages using a chemical cocktail termed 6TCF. We termed this phenomenon as iMT^[^ ^8^ ^]^ (**Figure** **1**A–C). The 6TCF cocktail comprises four key components with distinct molecular mechanisms: E616452 functions as a TGF‐β signaling inhibitor,^[^ ^16^ ^]^ tranylcypromine promotes H3K4 methylation,^[^ ^17^ ^]^ CHIR99021 serves as a WNT signaling activator,^[^ ^18^ ^]^ and forskolin (FSK) enhances intracellular cAMP levels^[^ ^19^ ^]^ (Figure 1A). Notably, the established iMT products maintained stable morphological and transcriptomic profiles even after the withdrawal of small molecules (SMs) (Figure 1A,B). To elucidate the dynamic molecular mechanism underlying this reprogramming process, we performed comprehensive multi‐omics profiling of iMT products across 10 distinct time points using RNA‐seq, ATAC‐seq and CUT&Tag technologies (Figure 1A; and Table S1, Supporting Information).

Comparative analysis revealed substantial alterations in RNA expression profiles between iMT products and control samples cultured without SMs (Figure S1C, Supporting Information). Gene expression heatmap analysis demonstrated the emergence of multilineage gene expression patterns during the iMT progression, with disappearance of MEF‐specific characteristics by day(D) 2 (Figure 1D; Figure S1D, Supporting Information). Through comprehensive evaluation of global gene and TF expression dynamics (Figure 1E; Figure S1E,F, Supporting Information),^[^ ^8^ ^]^ we categorized the 10 time points into four distinct phases: D0, Stage1, Stage2, and Stage3. Throughout this temporal progression, the exogenous SMs combination effectively activated developmental pathways and cell differentiation programs, concomitant with a progressive reduction in muscle and skeletal developmental signatures (Figure S2, Supporting Information). These observations were further corroborated by dynamic TF module analysis (Figure 1F).

To quantitatively evaluate cellular potency and plasticity, we performed entropy analysis following established methodologies.^[^ ^14^ ^]^ The average entropy of iMT cultures showed an initial decrease followed by a subsequent increase (Figure S3A, Supporting Information). Intriguingly, comprehensive gene set variation analysis (GSVA) of chromatin remodeling pathways revealed a dynamic trajectory, characterized by an initial upregulation in Stage1, subsequent downregulation in Stage2, and a secondary elevation in Stage3 (Figure S3B, Supporting Information). Gene ontology (GO) enrichment analysis of chromatin organization pathways further supported the extensive and dynamic epigenetic changes during cellular reprogramming (Figure S3C,D, Supporting Information). Notably, distinct histone modification pathways, particularly histone methylation and acetylation, demonstrated significantly elevated activity in stage1 (Figure S3E,F, Supporting Information). To gain deeper insights into the epigenetic features of Stage1, we conducted comprehensive analyses of key histone modifications (H3K4me1 and H3K4me3) and chromatin accessibility of D6 iMT cultures (Figure 1G–I). Our investigations revealed a significant enhancement of these positive epigenetic markers during Stage1, creating a permissive chromatin state conducive to the activation of lineage‐specific TFs (Figure S4A,B, Supporting Information). Furthermore, we identified Mll2/Kmt2c, a core component of the Set1/COMPASS methyltransferase complex, as a potential key regulator in this process. (Figure 1J–L). This enzyme plays an important role in promoting H3K4 methylation and indirectly facilitating to establish an open chromatin configuration. These collective findings strongly suggest that chromosomal epigenetic remodeling plays a pivotal role in stage1.

For a comprehensive multi‐omics analysis, we performed TF module analysis on both ATAC‐seq and H3K4me3 CUT&Tag datasets (Figure S5, Supporting Information), which revealed regulatory trends consistent with the transcriptomic data (Figure 1F). Notably, we observed a progressive upregulation of development‐associated TF modules over time, accompanied by a corresponding downregulation of muscle‐related modules (Figure S5A,B, Supporting Information). By integrating TF activity profiles across the three omics datasets, we identified 45 TFs—including Msx1, Twist1, and Foxd4—that were coordinately upregulated in stage1 at both epigenetic and transcriptional levels. GO enrichment analysis of these TFs demonstrated their predominant association with developmental regulatory pathways (Figure S5C,D, Supporting Information).

### 2.2 Mapping the iMT Atlas via scRNA‐seq

**Figure 2:**
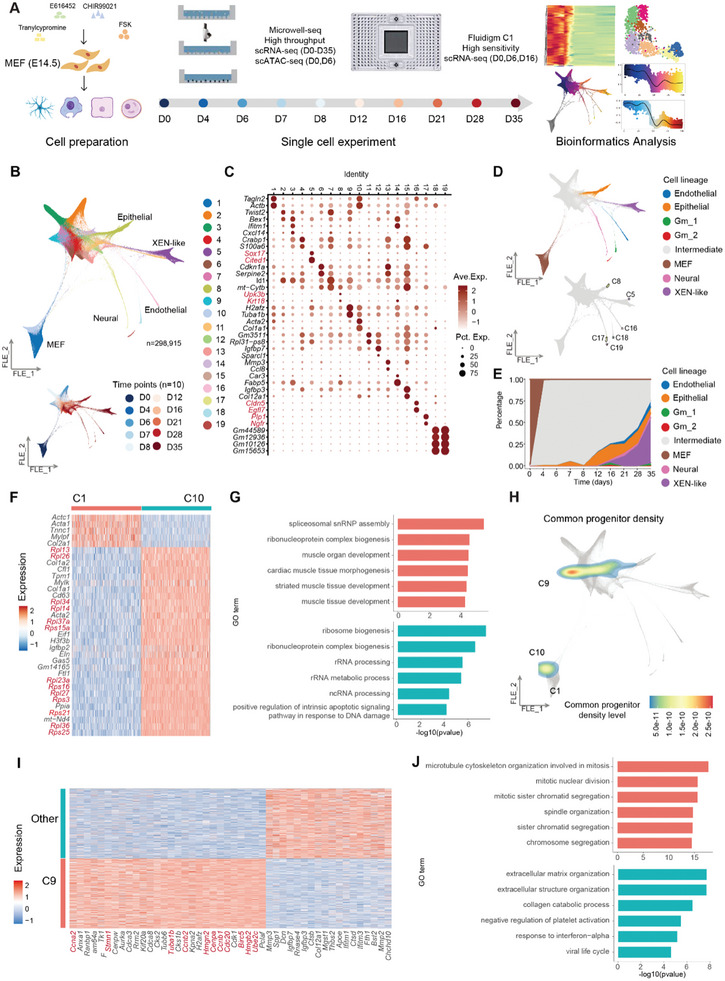
Mapping cell fates during iMT Process using scRNA‐seq. A) Overview of the single cell sequencing and bioinformatics workflow. B) FLE visualization of 298,915 single cells from Microwell‐seq, colored by cluster identities (top) and time points (bottom). C) A dotplot showing the scaled average expression levels of cell type‐specific marker genes (columns) in each cluster (rows). The size of the points represents the gene expression proportion, and the color represents the relative expression level. D) FLE visualization of 298,915 single cells from Microwell‐seq, colored by cell lineages (top) and terminal macrostates (bottom). E) A percentage stacked chart showing the distribution of cell lineages across ten time points. The x‐axis represents the time points, while the y‐axis shows the proportion of different cell types at specific time points. F) A heatmap showing the expression of signature genes (columns) of C1 and C10 (rows). G) Barcharts showing the representative GO terms enriched in marker genes of C1 (top, red) and C10 (bottom, blue). H) FLE visualization of 298,915 single cells from Microwell‐seq, colored by common ancestral density level. I) A heatmap showing the expression of signature genes (rows) of C9 and other intermediate cells (columns). J) Barcharts showing the representative GO terms enriched in marker genes of C9 (top, red) and other intermediate cells (bottom, blue).

To map iMT landscape, we employed two complementary approaches to construct scRNA‐seq library. Microwell‐seq was utilized to profile a broad spectrum of cell types with high throughput and compatibility, while the Fluidigm C1 platform was chosen for its higher sensitivity, enabling the capture of an average of 4000 genes per cell. We collected the sequencing data of 300344 cells across 10 time points during the iMT process: D0, D4, D6, D7, D8, D12, D16, D21, D28, and D35 (**Figure** **2**A). To investigate the dynamic changes in cell composition during iMT, we performed dimension reduction and leiden clustering, projecting 298915 cells from Microwell‐seq into a 2D force‐directed layout embedding (FLE) space. This analysis revealed 19 distinct cell clusters (Figure 2B,C; and Table S2, Supporting Information), which were further categorized into three major groups based on cellular identity: MEFs, intermediate cells, and terminal‐state cells (Figure 2D,E). Among these, six terminal cell clusters mainly distributed in Stage3 were identified (Figure S6A, Supporting Information), including XEN‐like cells (marked by *Sox17* and *Cited1*, Cluster(C) 5), endothelial cells (characterized by *Cldn5* and *Egf17*, C16), neural cells (identified by *Plp1* and *Ngfr*, C17), epithelial cells (marked by *Upk3b* and *Krt18*, C8), and two undefined cell populations with high levels of predicted genes and pseudogenes (C18 and C19) (Figure 2C–E; Figure S6B, Supporting Information). MEFs were further divided into two subsets: C1, expressing *Mgp* and enriched in muscle‐related genes, and C10, expressing *Tpm4* and showing higher expression of ribosomal protein (RP) genes (Figure 2F,G). Eleven intermediate cell clusters (C2, C3, C4, C6, C7, C9, C11, C12, C13, C14, and C15), primarily distributed in Stage1 and Stage2 (Figure 1C; Figure S6A, Supporting Information), were enriched in genes associated with embryonic development, cell motility and migration, and cell apoptosis (Figure S6B, Supporting Information). Additionally, cells captured using Fluidigm C1 platform were clustered into 8 groups with 4378 genes and 82255 confidently mapped reads per cell (Figure S7A–D, Supporting Information). Data from both platforms showed strong consistency (Figure S7E and Table S3, Supporting Information). To facilitate public access to the iMT data, we constructed a website available at https://bis.zju.edu.cn/iMT/.

To delineate the gradual process of trans‐differentiation at the single‐cell level, we utilized Waddington‐OT to reconstruct cellular trajectories by identifying ancestors, descendants, and their dynamic relationships through a time‐varying probability distribution. Notably, C10 characterized by elevated expression of ribosomal protein (RP) genes, exhibited higher contribution than C1 to the subsequent trans‐differentiation process (Figure 2H; Figure S6B, Supporting Information), suggesting distinct reprogramming potentials among MEF subtypes. In addition, C9 with high expression level of cell cycle‐related genes (Figure 2H–J), also showed significant ancestral potential. We therefore defined C9 as intermediate initial cells.

To determine the onset time of signaling pathways, we conducted GSVA to assess signaling pathways associated with forskolin, E616452 and CHIR99021 during iMT (Figure S8, Supporting Information). Our analysis revealed significant alterations in relevant signaling pathways during the early stage of trans‐differentiation. To further characterize stage‐specific transcriptomic features, we categorized 8000 highly variable genes into eight distinct modules based on their global expression patterns (Figure S9A,B, Supporting Information). Overall, we observed a sharp decline in MEF features (Module 2) alongside a gradual upregulation of lineage‐specific markers (Module 5 and Module 7) (Figure S9C,D, Supporting Information). Interestingly, two distinct high cell cycle activity phases were identified, corresponding to stage1 and early stage3, respectively (Figure 1C). This finding indicates that there is a periodic variation in cell proliferation during the iMT process.

### 2.3 Transition from MEFs into Intermediate Initial Cells

**Figure 3:**
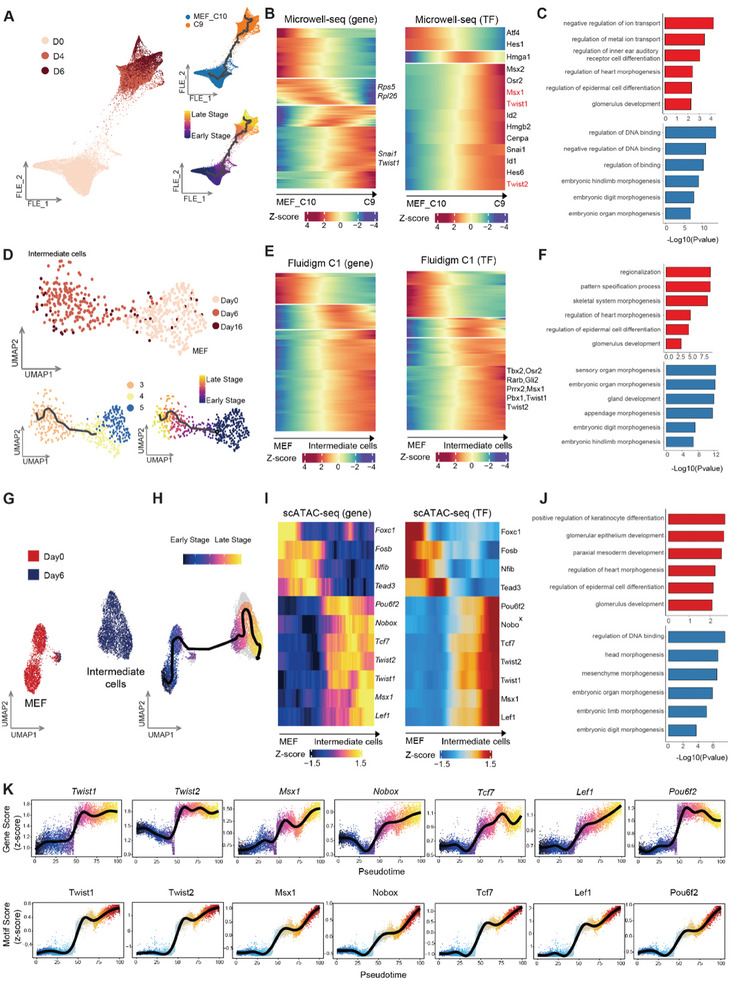
The initiation of reprogramming from MEFs to intermediate initial cells. A) FLE visualization of C10 (MEFs) and C9 (intermediate initial cells) from Microwell‐seq, colored by time points (left), cell identities (right top), and stages (right bottom). B) Heatmaps showing the expression levels of different genes (left) and TFs (right) (q.value < 0.001 & morans.I > 0.06) across stages along the trajectory. C) Barcharts illustrating enriched representative GO terms in marker TFs for the early (top, red) and late stages (bottom, blue) of the trajectory. D) UMAP visualization of MEFs (cluster4, 5) and intermediate initial cells (cluster3) from C1‐seq, colored by time points (top), cell identities (from Figure S7A, Supporting Information, bottom left), and stages (bottom right). E) Heatmaps showing the expression levels of representative genes (left) and TFs (right) (q.value < 0.001 & morans.I > 0.1) across stages along the trajectory. F) Barcharts illustrating enriched representative GO terms of marker TFs for the early (top, red) and late stages (bottom, blue) of the trajectory. G,H) UMAP visualization of scATAC‐seq profiles, colored by time points (G) and cell stages (H). I) Heatmaps showing the significant dynamic gene activity (left) and motif deviations score (right) along the MEFs to intermediate cells trajectory. J) Barcharts illustrating enriched representative GO terms in marker TFs for the early (top, red) and late (bottom, blue) stages of the trajectory of scATAC‐seq data. K) Gene activity of reprehensive genes (top) and motif deviation of reprehensive TFs (bottom) along the MEFs to intermediate cells trajectory of scATAC‐seq data.

First, we analyzed the trajectory from reprogramming‐capable MEFs (C10) to the intermediate initial state (C9) (**Figure** **3**A). Along the pseudotime trajectory, TFs associated with embryonic development accumulated, while those related to epidermal differentiation declined (Figure 3B,C; Figure S10, Supporting Information). Furthermore, we compared TF expression profiles between D6 cells and MEFs (Figure S11A, Supporting Information). A total of 58 TFs were upregulated, including Twist1, Tcf4, and Tfap2c, which are known to play roles in multi‐organ development. Conversely, TFs involved in skeletal morphogenesis were downregulated (Figure S11B,C, Supporting Information). Additionally, leveraging the exceptional sensitivity of the Fluidigm C1 platform, we detected a broader spectrum of relevant TFs (Figure 3D–F; Figure S11D and Table S4, Supporting Information).

To further explore the epigenetic changes from MEFs to intermediate initial cells, we collected chromatin accessibility data using Microwell‐based single‐cell ATAC sequencing (scATAC‐seq). Sequencing data of cells at D0 (n = 3232) and D6 (n = 4784) were obtained (Figure 3G,H). Along the pseudotime trajectory, we observed a significant increase in motif enrichment scores and gene scores for Twist1, Twist2, and Msx1, which are associated with multi‐lineage development. Conversely, those scores for Nfib and Tead3, which are related to epidermal differentiation, decreased along the pseudotime trajectory (Figure 3I–K; Figures S12 and S13, Supporting Information). Notably, the openness of TF motifs for cell lineage‐specific TFs, such as Gsx1 (pituitary),^[^ ^20^ ^]^ Tcf21 (stromal),^[^ ^21^ ^]^ Neurod1 (neural),^[^ ^22^ ^]^ Neurog1 (neural),^[^ ^23^ ^]^ Myod1 (muscle),^[^ ^24^ ^]^ and Myog (muscle), showed a gradual increase (Figure S13D, Supporting Information).

Overall, our findings demonstrated that the intermediate initial cells transition into a highly plastic and potent state, facilitating cell fate determination toward multiple lineages. Transcriptomic and epigenetic analyses revealed that development‐related TFs, including Twist1, Twist2, and Msx1 function as crucial activators in initiating chemical induced trans‐differentiation.

### 2.4 Trajectory Reconstruction from Intermediate Initial Cells to Terminal States

**Figure 4:**
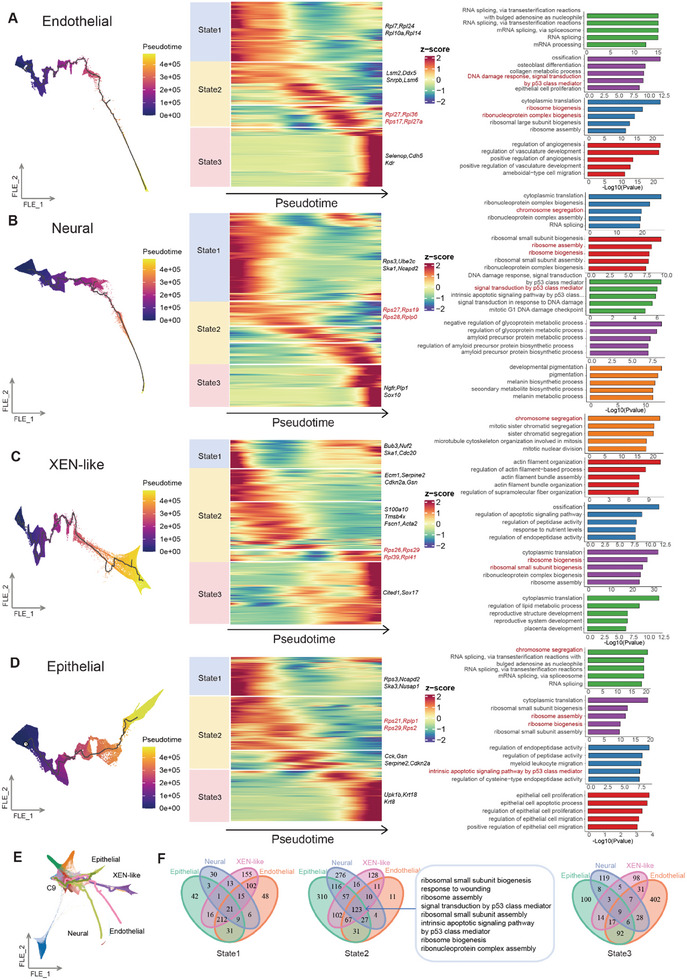
Reconstruction of terminal cell reprogramming trajectory. A**–**D) Reconstruction of terminal cell reprogramming trajectories from C9 to endothelial cells (A), C9 to neural cells (B), C9 to XEN‐like cells (C), and C9 to epithelial cells (D), and the corresponding gene modules analyses of these cell trajectories. FLE visualization of the cell trajectories, colored by pseudotime (left). Heatmaps showing the gene modules at different pseudotime in the corresponding cell trajectories (middle). Barcharts showing representative enriched GO pathways enriched in the corresponding gene modules (right). E) A FLE visualization of four trajectories from C9 to terminal cell types, colored by cluster identities. F) Venn diagrams illustrates the differences and commonalities in enriched GO terms among four lineages of gene modules in the initial (left), intermediate (middle), and final (right) states of the reprogramming trajectory.

Given the high ancestral potential of C9 (Figure 2H), we hypothesized that terminal cells originated from cells in this cluster. To explore these transitions, we constructed four distinct trajectories between C9 and terminal cell types—including XEN like cells, endothelial cells, neural cells, and epithelial cells—using Monocle 3 (**Figure** **4**A–E).^[^ ^25^ ^]^ For each trajectory, genes were categorized into multiple modules based on the correlation between their expression levels and pseudotime (Figure 4A–E; and Table S5, Supporting Information). These modules were further classified into three distinct states. GO analysis revealed that State1 was primarily enriched in cell cycle‐related pathways, whereas State2 showed enrichment in ribosome biogenesis pathways. Furthermore, we observed a gradual accumulation of lineage‐specific GO terms in state3, delineating the distinct cellular dynamics from intermediate initial cells to diverse end states.

We subsequently integrated the GO terms across the four trajectories and found that state2 shared the most terms (Figure 4F). Through comprehensive analysis of dynamic RP scores, we demonstrated that elevated expression of RP genes represents a conserved feature of state2 across all four trajectories (Figure S14, Supporting Information). Moreover, by clustering 8000 highly variable genes into eight distinct modules (Figure S15, Supporting Information), we identified that Module 7, which is significantly enriched in ribosome‐related pathways, shows prominent accumulation in state2 followed by a sharp decrease in terminal cells. Importantly, this pattern of high RP expression has been consistently observed during the development of both ectoderm and endoderm lineages^[^ ^26^ ^]^ (Figure S16, Supporting Information). These findings collectively suggested that the heightened expression of RP genes may play a key role in the process of cell fate determination.

### 2.5 The Characteristics of Terminal Cells in iMT

**Figure 5:**
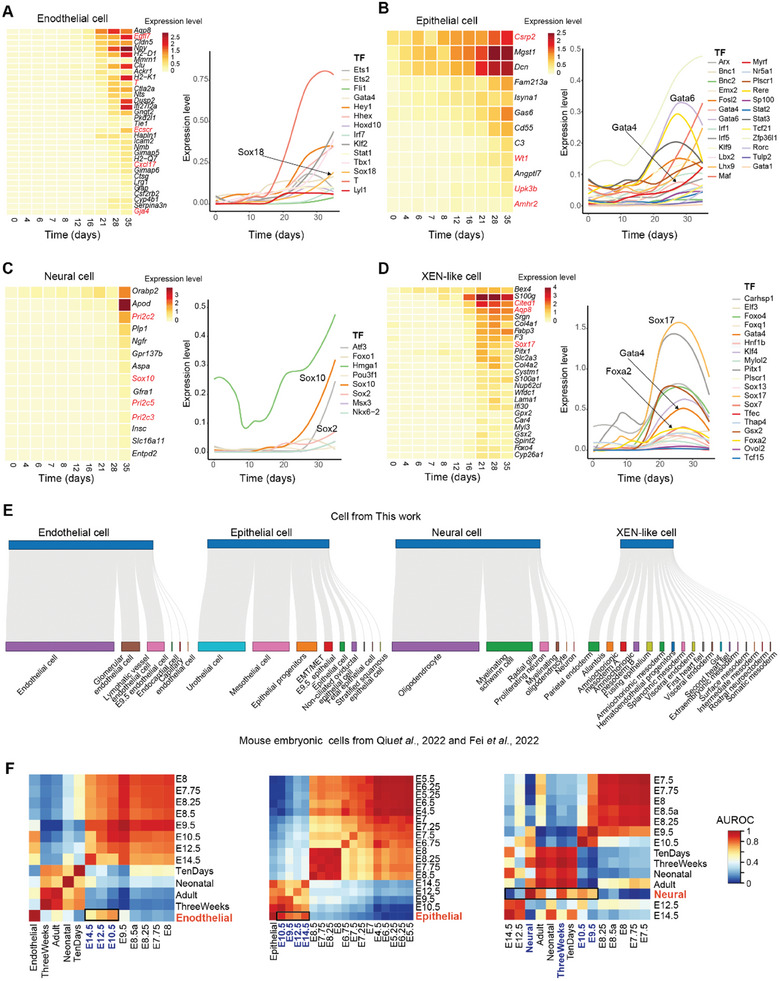
The characteristics of terminal macrostates during trans‐differentiation. A**–**D) Heatmaps and line charts showing dynamic expression of lineage‐specific genes (left) and TFs (right) in endothelial cells (A), epithelial cells (B), neural cells (C), and XEN‐like cells (D), respectively. E) Sankey plots showing correspondence relationships between terminal cell types in our work (top) and mouse embryonic cell types in previous studies (bottom).^[^ ^15^, ^26^ ^]^ F) Heatmaps showing correspondence relationships between terminal cell types in our work and mouse embryonic cells from different stages in previous studies (see Experimental Section for details).

To elucidate the molecular and cellular characteristics of terminal cells, we conducted a comprehensive analysis to identify potential driver genes and transcription factors (TFs) associated with terminal cell states. By correlating the expression levels of all genes with fate probabilities derived from CellRank,^[^ ^27^ ^]^ we identified a cascade of gene activation events in terminal cells at the single‐cell level (**Figure** **5**A–D). We excluded C18 and C19 due to their high levels of predicted pseudogenes.

Among the identified driver genes, several blood vessel development‐related genes, including *Gja4*, *T*, *Tie1*, *Ecscr*, *Cxcl17*, and *Egfl7*, were specifically activated in endothelial cells. In epithelial cells, we observed high expression of the urothelium‐specific marker *Upk3b*, along with the mesenchymal‐to‐epithelial transition (MET)‐related gene *Amhr2*,^[^ ^28^ ^]^ the development and cellular differentiation‐related gene *Csrp2*, and the epithelial–mesenchymal balance maintenance gene *Wt1*.^[^ ^29^ ^]^ In neural cells, genes associated with the positive regulation of neural precursor cell proliferation, such as *Anger*, *Prl2c2*, *Sox10*, *Aspa*, and Proliferin (*Prl2c2*, *Prl2c5*, and *Prl2c3*), were prominently activated. Additionally, the expression levels of *Cited1*, *Sox17*, and *Aqp8* suggested an extraembryonic cell fate decision at D21 (Figure 5A–D).

Among the identified driver TFs, terminal macrostates exhibited SOX and GATA TF activities, including Sox18 in endothelial cells, Sox10/2 in neural cells, and Gata6/4 in epithelial cells. Notably, Sox17, Gata4, and Foxa2 were sequentially activated and reached peak expression levels in a stepwise manner in XEN‐like cells, consistent with previous findings.^[^ ^30^ ^]^ Additionally, key regulators Stat3 and Sox7, which are known to facilitate rapid chemical reprogramming,^[^ ^31^ ^]^ were significantly upregulated in epithelial and XEN‐like cells (Figure 5A–D). To identify common transcriptional activators, we performed promoter region enrichment analysis of driver TFs from each terminal cell type and identified motifs with the highest Normalized Enrichment Score (NES). This analysis predicted TFs including Elf3, Srf, and Zfb384 as potential common regulators (Figure S17A, Supporting Information). In addition, we observed a global increase in chromatin accessibility for both commom and lineage‐specific TFs in Stage3 (Figure S17B–F, Supporting Information). Thus, the identified TFs provided valuable resources for elucidating the key regulatory factors underlying chemical induced trans‐differentiation.

We performed an integrated analysis of scRNA‐seq data for iMT cells and embryonic cells from embryonic (E)10.5 to E14.5.^[^ ^26^ ^]^ Our findings revealed that endothelial, neural, and epithelial cells in iMT cultures could be successfully integrated with their corresponding embryonic counterparts. In contrast, all intermediate state cells failed to integrate with somatic cells in vivo, underscoring significant divergence trajectories between iMT and natural development processes (Figure S18, Supporting Information). To further investigate these relationships, we employed scPoli,^[^ ^32^ ^]^ a semi‐supervised conditional deep generative model, to construct a query‐to‐reference map between terminal macrostates and mouse embryonic cell types from E9.5 to E14.5^[^ ^15^, ^25^ ^]^ and develop cell lineage‐specific reference models (Figure 5E; Figure S19, Supporting Information). In this study, MEFs for iMT were derived from E14.5 embryos. As anticipated, 62.4% of MEFs showed consistency with stromal cells from E14.5 (Figure S20A, Supporting Information). Surprisingly, terminal cells exhibited strong correlations with corresponding cells from earlier developmental stages than E14.5, suggesting that endothelial, epithelial, neural and XEN‐like cells from iMT transitioned to a more youthful state compared to their initial mode (Figure 5F; Figure S20B–E, Supporting Information). This observation was further supported by gene expression profiles, which showed greater similarity between trans‐differentiation cells and their corresponding cells from earlier embryonic stages (Figure S21A–C, Supporting Information), reinforcing the concept of cellular rejuvenation through trans‐differentiation.

### 2.6 Comparisons of iMT with Other Cell Fate Determination Processes

**Figure 6:**
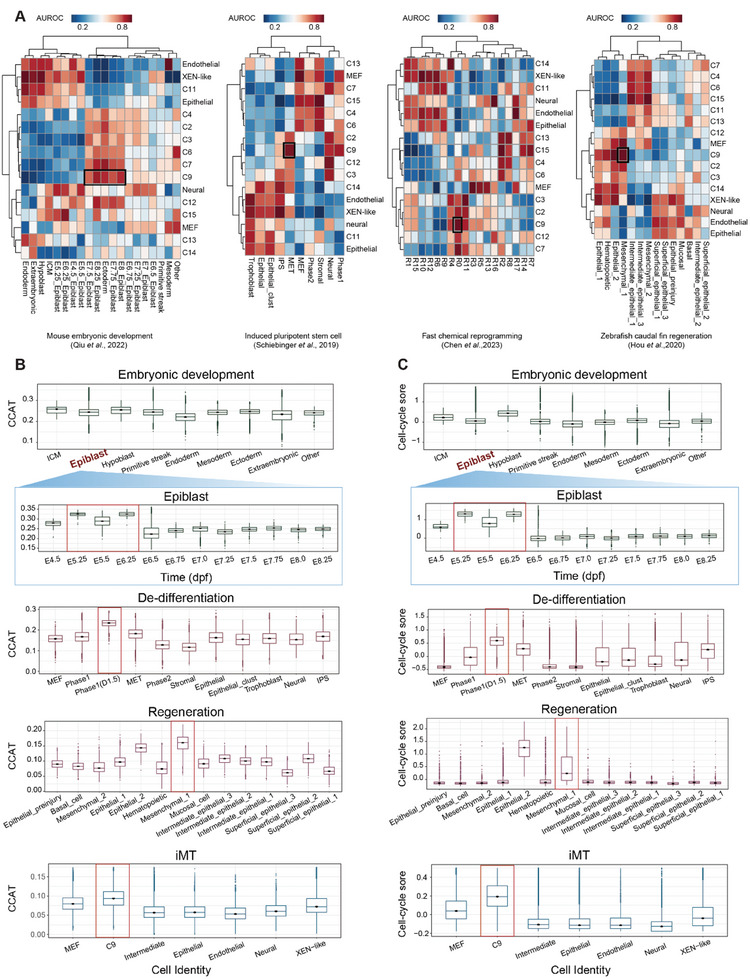
Comparison between iMT, de‐differentiation, embryonic development and regeneration processes. A) Heatmaps showing the cell type correlation between trans‐differentiation process (this work, rows) and other four cell fate determination process (mouse embryonic development,^[^ ^26^ ^]^ de‐differentiation^[^ ^3^ ^]^ and fast chemical reprogramming^[^ ^31^ ^]^ and zebrafish fin regeneration process,^[^ ^13^ ^]^ columns) from left to right. B) Boxplots showing the distribution of the CCAT entropy values across different cell types in embryonic development,^[^ ^26^ ^]^ de‐differentiation,^[^ ^3^ ^]^ regeneration,^[^ ^13^ ^]^ and trans‐differentiation (this work) from top to bottom. Significant changes in the CCAT entropy of the epiblast from E4.5 to E8.75 are highlighted. C) Boxplots showing the distribution of cell‐cycle scores across different cell types in embryonic development,^[^ ^26^ ^]^ de‐differentiation,^[^ ^3^ ^]^ regeneration,^[^ ^13^ ^]^ and trans‐differentiation (this work) from top to bottom. Significant changes in the cell‐cycle score of the epiblast from E4.5 to E8.75 are highlighted.

Cell fate determinations encompasses a variety of processes, including development^[^ ^26^ ^]^ and regeneration^[^ ^13^ ^]^ in vivo, as well as enforced de‐differentiation^[^ ^3^, ^31^ ^]^ and trans‐differentiation in vitro.^[^ ^33^ ^]^ In our previous analysis, we identified that Msx1 plays a crucial role in iMT process (Figure 3). Similar observations have been reported in human fibroblast reprogramming^[^ ^12^ ^]^ and axolotls limb regeneration.^[^ ^34^, ^35^, ^36^ ^]^ Based on these findings, we hypothesized that these cell fate determination processes might share analogous cell states and molecular events. To explore this, we employed MetaNeighbour^[^ ^37^ ^]^ to quantitatively assess similarities among cell types involved in iMT and other classic cell fate determination processes. Our analysis revealed that multiple cell types in iMT exhibited significant similarities with those identified in other processes. Notably, we identified clusters highly similar to C9 across all processes, which we termed C9‐like (**Figure** **6**A).

We then conducted comparative analyses between iMT and other cell fate decision processes (Figures S22–S24, Supporting Information). In comparison to the de‐differentiation of somatic cells,^[^ ^3^ ^]^ we found that the expression pattern of C9 is highly consistent with Phase 1 in mouse iPS and with R8 and R9 in human iPS processes. Both Phase1 and R8/R9 represent critical intermediate plastic states during the early stages of reprogramming, characterized by elevated levels of cell cycle and mitosis activity (Figure S22E,F, Supporting Information). Similarly, R0 in rapid chemical reprogramming displayed analogous characteristics as C9. Furthermore, *Larp1*, *Slc17a5*, and *Prkaa1*, which have been previously implicated in promoting a diapause‐like state in chemical reprogramming, exhibited increased gene expression levels at D21‐D28 (Figure S23, Supporting Information). Interestingly, we also observed C9‐like cells (Mesenchymal1) in the fin regeneration process, characterized by high entropy and elevated expression of *Kpna2* and *Mad2l1* (Figure S24, Supporting Information). These findings indicated that the states of C9 and C9‐like cells are common and must‐pass in cell fate determination processes. We have defined these states as metastable cell states.

To further elucidate the characteristics of common metastable cell states, we observed that entropy and cell cycle activities undergo synchronized dynamic changes during cell fate determination processes (Figure S25, Supporting Information). Additionally, we found that C9 and C9‐like cells exhibited a sharp increase in entropy and cell cycle scores (Figure 6B,C). By systematically quantifying the sources of elevated entropy values in metastable cell states, we identified significantly upregulated expression of Rp genes, as well as genes regulating protein localization to chromosome and telomere (Figure S26, Supporting Information). These features collectively provided the necessary energy and materials to support cell division (Figure 6C; Figure S25B, Supporting Information).

Collectively, through in‐depth time‐course analysis, we have uncovered shared metastable states during cell fate transitions, which are characterized by pronounced increases in entropy and cell cycle activity. This study deepens our understanding of the potential epigenetic and transcriptomic regulators involved in trans‐differentiation, and provides valuable insights into molecular mechanisms that may govern the dynamics of cell fate determination.

## 3 Discussion

**Figure 7:**
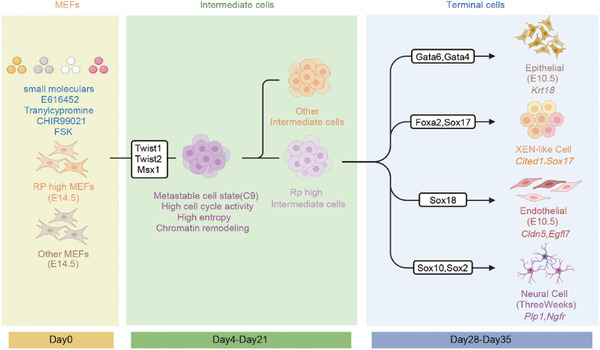
A schematic of the iMT process.

In our previous study, we demonstrated that MEFs can be induced to trans‐differentiate into multiple somatic lineages using a combination of four chemicals, collectively known as 6TCF. To further investigate the trans‐differentiation process through chemical reprogramming, we performed a multiomics analysis of cells undergoing iMT at a global level. Our findings revealed that SMs play a key role in facilitating chromosomal remodeling, which enhances chromatin accessibility. This remodeling phase is followed by a consolidation period marked by a transient reduction in gene expression levels. In the final stages, the expression of lineage‐specific genes is upregulated, effectively eliminating the MEF characteristics and driving the cells toward their new identity (**Figure** **7**). We propose that both intrinsic TF fluctuation and extrinsic micro‐environmental cues may lead to the diversification of multi‐lineage cell fate decision.

To elucidate the molecular mechanisms governing the cell fate decisions during iMT, we generated a comprehensive single‐cell dataset throughout the entire trans‐differentiation process using two distinct scRNA‐seq approaches. Fluidigm C1 offers high sensitivity, enabling the detection of low‐abundance transcripts. However, due to its high cost and low throughput, it is not ideal for capturing the full cellular dynamics of the iMT process. To address this limitation, we also employed Microwell‐seq, a low‐cost, high‐throughput technology. Together, these two methods provide a balanced approach, combining sensitivity and throughput to profile the iMT atlas effectively. Using this integrated strategy, we identified 19 distinct cell types, revealing trajectories from mouse embryonic fibroblasts (MEFs) to fully trans‐differentiated cells, including intermediate states and terminal macrostates.

The methylation of H3K4 is positively correlated with chromosome openness and gene expression. LSD1 specifically demethylates histone H3 lysine 4, particularly H3K4me1/2.^[^ ^38^ ^]^ Tranylcypromine (T) plays a critical role by directly inhibiting LSD1, thereby inhibiting H3K4 demethylation.^[^ ^39^ ^]^ We observed an elevated peak number of H3K4me1 and H3K4me3 at stage2 of iMT. This will lead to a widespread increase in chromosome openness during this period. In addition, H3K4 methylation is deposited by SET1/COMPASS complexes.^[^ ^40^ ^]^ Mll2/Kmt2c, a component of the SET1/COMPASS complexes, may also play a role in increasing chromosome accessibility. These conclusions suggested that Tranylcypromine indirectly contribute to broadly chromatin openness during iMT process.

Notably, we revealed a population of intermediate initial cells (C9) as a mandatory transitional state during the iMT process. These cells are characterized by high expression of multiple TFs, high cell cycle activity, and high entropy. According to previous research, MEFs exhibit higher cell cycle activity and high potency compared to adult cells.^[^ ^15^ ^]^ These features may account for the higher reprogramming efficiency of MEFs relative to adult cells.^[^ ^8^ ^]^ All terminal cells originate from this intermediate state, which exhibits a uniform gene expression profile and lacks lineage‐specific features (Figure S27C, Supporting Information). SMs prompt the openness of regulatory regions of the genome and increase the expression of key TFs such as Twist1, Twist2, and Msx1, thereby restoring MEF cellular plasticity to intermediate priming state. Notably, while Twist1 upregulation has been observed in SMs‐induced directional reprogramming study,^[^ ^41^ ^]^ the combined upregulation of Twist1, Twist2, and Msx1 is reported for the first time in process of trans‐differentiation.

As the iMT process progresses into the intermediate stages (stage3), cell division and cell fate transition require substantial protein synthesis, which is supported by ribosome biogenesis. This activity is essential for the reprogramming of priming cells into diverse somatic lineages. Subsequently, lineage‐specific TFs are gradually activated. Drawing on Waddington's epigenetic landscape theory,^[^ ^42^ ^]^ we propose that appropriate attractor combinations and dosage of attractors guide intermediate cells into the corresponding attractor basins. As the expression pattern stabilize in the attractors, cells begin to transit into a diverse array of cell types representing the entire spectrum of embryonic layers in vitro, including endothelial, epithelial, XEN‐like, and neural cell types. Moreover, directional trans‐differentiation efficiency could be improved by modified stepwise chemical treatment in our previous study.^[^ ^8^ ^]^ Interestingly, we revealed through scPoli and Metaneighbor analyses that reprogrammed cells can be reverted to a state earlier than E14.5. However, this phenomenon is currently based primarily on transcriptomic characteristics. Further functional validation is required to assess the potential of these trans‐differentiation cells. Additionally, due to the reset of the MEFs’ epigenetic profile, some cells fail to be correctly guided into their corresponding attractor basins, leading to reprogramming failure (Figure S27, Supporting Information).

To gain a deeper understanding of the iMT process, we systematically compared it with several well‐established cell fate determination processes, including embryonic development, de‐differentiation, and regeneration. Through cross‐process and cross‐dataset analyses, we identified key features of diverse cellular destinies. Notably, a initial high‐potential state (C9 and C9‐like cells), characterized by elevated entropy and cell cycle, was found to coexist across different cell fate decision processes before cells transition into stable cell types. The subsequent increase in entropy suggests that certain cells may have entered a reprogrammable, more rejuvenated state. This state is responsive to homeostatic signals by attractor regulatory networks. This process mirrors key aspects of early embryonic development, where cells undergo significant programming before stabilizing into defined cell types.^[^ ^15^ ^]^

We have established a dynamic atlas of iMT process over 35 days, systematically characterized the intermediate initial state (C9) and terminal cells. We identified commonalities between iMT and other cell fate determination processes. Future optimization of experimental workflow is beneficial to generate directive reprogrammed cells, and to improve the efficiency of adult cells reprogramming. Further functional validations of iMT products are also needed for potential therapeutic applications. Importantly, the efficiency and lineage preferences of cell trans‐differentiation may differ between mouse and human cells, meaning the current conclusions may not directly apply to human systems. Future studies should investigate the applicability of our model across different cell lines and species.

In summary, our study provides valuable resources for identifying key regulatory factors involved in trans‐differentiation. We emphasized the role of SMs in regulating this process and deepened our understanding of the complex mechanisms governing cell fate decision. These findings have significant potential for advancing regenerative medicine.

## 4 Experimental Section

### Ethics Statement

All experiments performed in this study were approved by the Animal Ethics Committee of Zhejiang University (ZJU20220202). All experiments conformed to the relevant regulatory standards at Zhejiang University Laboratory Animal Center.

### Cell Culture and Trans‐Differentiation for MEFs

MEFs were isolated from E14.5 C57BL/6J mouse embryos. Head, vertebral column, dorsal root ganglia, and all internal organs were removed and discarded. The remaining embryonic tissue was manually cut into pieces and incubated in 0.25% trypsin for 5 min. Cells from embryos were plated onto a 15 cm tissue culture dish in MEF medium contains DMEM (Life Technologies) supplemented with 15% FBS (Gibco), 2 mm GlutaMax supplement (Life Technologies), 1% nonessential amino acids (Life Technologies), 0.1 mm β‐mercaptoethanol (Sigma). A total of 9 embryos were used to establish MEF cell lines. MEFs of 2–4 passage were used for iMT experiment. MEFs were seeded at a density of 20000 cells per well in six‐well plate. The next day, MEF medium was replaced with iMT media contains DMEM (Life Technologies) supplemented with 15% FBS (Gibco), 2 mm GlutaMax supplement (Life Technologies), 1% nonessential amino acids (Life Technologies), 0.1 mm β‐mercaptoethanol (Sigma), and 40 ng mL^−1^ basic fibroblast growth factor (bFGF, Stemgent), along with the chemical components of 6TCF (5 µm E616452, 5 µm Tranylcypromine, 10 µm CHIR99021, 10 µm FSK). Media were changed every 4 days. D0 MEF cells were harvested before iMT medium changed. Cells were harvested respectively on day 2, 4, 6, 7, 8, 12, 16, 21, 28, and 35 in chemical cocktail medium, with two wells collected at each time point for each sample. To verify the stability of the trans‐differentiation products at day 35, the cells were cultured with iMT medium without SMs for an additional three days.

### Cell Preparation

After digestion with 0.25% Tryisin for 5 min, cells were centrifuged at 300 × g for 5 min at 4 °C, then re‐suspended in 3 mL of cold DPBS. For Microwell‐seq, cells were diluted to 1 × 10^5^ cells mL^−1^ in DPBS with 2 mm EDTA. For Microwell ATAC‐seq, cells were suspended in 1 mL Nuclear Permeabilization Buffer (5% BSA, 0.2% IGEPAL CA‐630, 1 mm DTT, 1 × Roche Complete Protease Inhibitor in DPBS) and followed by incubation for 6 min to isolate the nuclei. The suspension was transferred to 15 mL centrifuge tubes pre‐filled with 5 mL wash buffer (1% BSA, 2 mm EDTA in DPBS), centrifuged at 500 × g for 5 min at 4 °C, and re‐suspended in 1 × DPBS.

### Microwell ATAC‐seq—Synthesis of Barcoded Beads

Magnetic beads coated with carboxyl groups were provided by Suzhou Knowledge & Benefit Sphere Tech (diameter 28 µm used in Microwell ATAC‐seq). The barcoded oligonucleotides on the surface of the beads were synthesized by three rounds of split‐pool. All the sequences used were listed in Table S6 (Supporting Information). For the detailed process of synthesizing magnetic beads, please refer to the previously published article.^[^ ^43^ ^]^

### Microwell ATAC‐seq—Tn5/pG‐Tn5 Tagging Plate Preparation

Add 2 µL of 100 µm Tn5_primerA (dissolved in annealing buffer), 1 µL of 100 µm indexed Tn5_primerB (encoding a unique barcode, 96 well‐specific, dissolved in annealing buffer) and 1 µL of 100 µm Tn5_primerC (dissolved in annealing buffer) to each well of 96‐well nuclease‐free plates, mix them and record the order. The sequence information was included in Table S5 (Supporting Information). The annealing program was as follows: 95 °C for 3 min; 75 °C for 10 min; 60 °C for 10 min; 50 °C for 10 min; 40 °C for 10 min, 25 °C for 30 min with a temperature ramp of −0.1 °C /s during the cooling process and a final 4 °C hold. Fifty microliters of TruePrep Tagment Enzyme (Vazyme, S111‐01/02) or Hyperactive pG‐Tn5 Transposase for CUT&Tag (Vazyme, S602‐01/02) were mixed with 160 µL of coupling buffer, and add 1.75 µL of mix to each well of a new 96‐well plate. After adapter annealing as described above, 1 µL annealing mix was aspirated from each well of the annealing plate and added to each well of the new plate correspondingly. The plate was fixed on rotary mixer (10 rpm) and incubated for 10 min at room temperature and followed by incubating the plate at 30 °C in the thermal cycler for 1 h. Then 10 µL of storage buffer was added to each well of the plates, and divided the final mix into other new plates, each well 2 µL, correspondingly. The Tn5/pG‐Tn5 tagging plate could be sealed and stored them at −20 °C for up to one year.

### Microwell ATAC‐seq—Preparation of Barcoded Beads

Before loading, the beads were washed twice with RSB buffer (10 mm Tris‐HCl pH 7.5, 10 mm NaCl, 3 mm MgCl_2_), and suspended in 50 µL beads anneal buffer 1 × Blue buffer [10 mm Tris‐HCl pH 7.9, 10 mm MgCl_2_, 50 mm NaCl, 1 mm DTT), 1 × PCR Enhancer (Vazyme), 5% PEG‐8000 (Sigma), 10 µm bridge oligos (Table S7, Supporting Information). The mixture in each tube was then evenly distributed to a nuclease‐free 8‐strip tube with 12.5 µL per well. The annealing program was as follows: 95 °C for 3 min; 65 °C for 1 min; 60 °C for 1 min; 50 °C for 1 min; 40 °C for 1 min with a temperature ramp of ‐0.1 °C/s during the cooling process, and a final 37 °C hold. Then the mixture was incubated at 37 °C on a rotary mixer (10 rpm) for 1 h. The beads were collected in 2 × SSC (Invitrogen, diluted in DEPC treated water) and washed with 200 µL TE‐SDS (0.5% SDS in TE), 200 µL 10 mm Tris‐HCl pH 8.0, and suspended in 500 µL DPBS‐E (2 mm EDTA in 1 × DPBS).

### Microwell ATAC‐seq—Tn5 Tagging of Nuclei

Permeabilized nuclei were counted using a hemocytometer and split into a 96‐well plate. For each well, 2.5 × 10^4^ nuclei in 2 µL DPBS, 2.5 µL ddH_2_O, 2.5 µL 4 × TD buffer [132 mm Tris‐HCl pH 8.0 (Invitrogen), 264 mm KOAc, 40 mm Mg(OAc)_2_, 64% N, N‐dimethylformamide in DEPC treated water], 1 µL 0.1% Digitonin and 2 µL 2 × TTE mix were added. Then the tagmentation was carried out at 37 °C for 1 h, and 10 µL 40 mm EDTA was added to each well. Next, the 96‐well plate was incubated at 37 °C for 10 min to completely stop the tagmentation.

### Microwell ATAC‐seq—Nucleus Collection and Lysis

Nuclei were collected, re‐suspended in ice‐cold wash buffer, and loaded on a microwell plate. The plate was then placed on a magnet, with beads being quickly shaken into the microwells. Excess beads were washed away slowly. Lysis buffer [1% SDS, 1 × Blue buffer, 5% PEG‐8000, 1 × PCR Enhancer (Vazyme), 10 mm EDTA, 1 mg mL^−1^ protease K (Sangon Biotech) in DEPC treated water] was added to lysis nuclei and removed after incubation for 30 min at 37 °C. Beads were collected and transferred to an RNase‐free tube, washed with 2 × SSC and 50 mm Tris‐HCl (pH 8.0).

### Microwell ATAC‐seq—Ligation

For ligation, beads were re‐suspended in ligation solution [1 × T4 ligase buffer, 5% PEG‐8000, 20 U T4 ligase (NEB, M0202)] and incubated at room temperature for 1 h on a rotary mixer (10 rpm). After ligation, beads were washed with 50 mm Tris‐HCl (pH 8.0) and suspended in the gapfill mix [1 × Blue buffer, 1 × PCR enhancer, 5% PEG‐8000, 1 mm dNTP and 6.25 U Klenow (NEB)], and incubated at 37 °C for 1 h on a rotary mixer (10 rpm).

### Microwell ATAC‐seq—Second Strand Synthesis

Beads were treated with 0.1 m NaOH and washed twice with 1 mL TE‐TW and 200 µL 10 mm Tris‐HCl (pH 8.0) before beads were re‐suspended in the second strand synthesis solution [1 × NEBuffer 2 (NEB, B7002S), 1 × PCR enhancer, 1 mm dNTP, 2 mm MgCl_2_, 4 µm i7 adaptor, 1 U 504 polymerase (Vazyme)]. The PCR program was as follows: 95 °C for 1 min; 5 cycles of 55 °C for 3 min, and 72 °C for 1 min; 72 °C for 5 min and 10 °C hold. After the second strand synthesis, beads were washed with 200 µL of TE‐SDS, 200 µL of TE‐TW, and 200 µL of 10 mm Tris‐HCl pH 8.0. After that, beads were re‐suspended in 50 µL exonuclease I mix and incubated at 37 °C on a rotary mixer (10 rpm) for 15 min to remove redundant beads oligonucleotides.

### Microwell ATAC‐seq—Library Preparation

After exonuclease digestion, beads were washed once with TE‐SDS, TE‐TW, and 10 mm Tris‐HCl pH 8.0. Beads were re‐suspended in PCR A mix [1 × HiFi HotStart Readymix (Kapa Biosystems), 0.2 mm MGI‐P5 primer, 0.4 mm MGI 2100 primer, 0.2 mm MGI‐P7 primer]. The PCR program was as follows: 95 °C for 5 min; 4 cycles of 98 °C 30 s, 60 °C 60 s, and 72 °C 1 min; 72 °C 3 min and 10 °C hold. After pooling all PCR products, 50 µL PCR mix B (1 × HiFi HotStart Readymix, 0.2 mm MGI 2100 in ddH2O) primer was added to each DNA sample. The PCR program was as follows: 95 °C for 3 min; 2 cycles of 98 °C for 30 s, 60 °C for 30 s, and 72 °C for 1 min; 12 cycles of 98 °C for 20 s, 65 °C for 30 s, and 72 °C for 1 min; 72 °C for 3 min; and 10 °C hold. To eliminate primer dimers and large fragments, 0.5 × & 0.5 × VAHTS DNA Clean Beads (Vazyme) were used to purify the library. Finally, the samples were subjected to sequencing on the MGI DNBSEQ‐T7. The protocol provided by VAHTS Circularization Kit for MGI (Vazyme) was applied to obtain the single‐stranded circular DNA available for DNB (DNA Nanoball) generation. The official read 1 sequencing primers were also replaced with the custom 2.0 read 1 sequencing primers 1&2 (The sequences information is included in Table S6, Supporting Information) to ensure the completion of the sequencing.

### Microwell‐seq

Single‐cell cDNA libraries were constructed according to the previously described Microwell‐seq protocol.^[^ ^43^ ^]^ The primers were provided in Table S8 (Supporting Information). The microwell device and the barcoded beads were pre‐prepared. After the single cells were collected and lysed, RNA was captured by beads and then reverse‐transcribed to cDNA, and exonuclease I was used to remove redundant beads oligonucleotides. After cDNA amplification, the customized transposase that carries two identical insertion sequences was utilized for the fragmentation of cDNA libraries, and fragments that contain the 3′ end of transcripts would be specifically amplified. cDNA library and sequence library were constructed for each cell sample separately. Each sequence library had unique index. Sequence libraries from different time points were pooled and sequenced on Illumina HiSeq X Ten with an average depth of 180 million reads per library. Sequencing process, including sample pooling, were operated by service providers (Annoroad Gene). For MGI sequencing, sequence libraries were pooled and applied the protocol provided by VAHTS Circularization Kit for MGI (Vazyme) to obtain single‐stranded circular cDNA available for DNB (DNA Nanoball) generation. The official read 1 sequencing primers were also replaced with the Read1 SeqA‐GT primer (for Illumina) or custom 2.0 read 1 sequencing primer 1&2 (for MGI) to ensure the completion of the sequencing. For Fluidigm C1 sequencing, the same single‐cell capture and library construction protocols were used as described previously.^[^ ^44^ ^]^ Samples obtained from HT IFCs were used to build sequence libraries with Nextera XT DNA Library Preparation Kit (Illumina). The samples were sequenced using the Illumina Hiseq platform with an average depth of 500 million reads per library.

### RNA‐seq

Total RNA was isolated using the FastPure Cell/Tissue Total RNA Isolation Kit V2 (Vazyme, RC112‐01) according to the manufacturer's instructions. Subsequently, add 2 µL of RNA with a total amount of ≈100–400 ng to a PCR tube containing 1 µL of dNTP and 1 µL of TSO‐oligodT. Incubate the PCR tube at 65 °C for 5 min and then place it immediately on ice for 2 min. 0.4 µL of ddH_2_O, 2 µL of 5 × PrimeScript II Buffer (Takara), 2 µL of Betaine (Sigma), 0.5 µL of 100 mm DTT, 0.15 µL of 0.4 m MgCl_2_, 0.2 µL of 50 mm TSO LNA primer, 0.25 µL of RNase inhibitor (Vazyme), and 0.5 µL of PrimeScript II RTase (Takara) were added. The reaction was gently mixed and carried out in a PCR thermocycler programmed as follows: 42 °C 120 min, 70 °C, 10 min; 10 °C hold. Following the reaction, a PCR amplification mix was prepared with 12.5 µL of 2 × HiFi HotStart ReadyMix, 1 µL of TSO‐PCR primer (10 µm), and 1.5 µL of ddH_2_O. The PCR program was as follows: 95 °C for 3 min; 7–16 cycles (adjust cycles based on RNA input) of 98 °C for 30 s, 65 °C for 15 s, and 72 °C for 6 min; 72 °C for 5 min; and 10 °C hold. Purification was then performed using 0.9 × of VAHTS DNA Clean Beads according to the manufacturer's instructions. Sequencing library construction was using TruePrep DNA Library Prep Kit for MGI (Vazyme, TDM503‐01) according to the manufacturer's instructions.

### Cut&Tag seq

Concanavalin A (ConA) coated magnetic beads (Vazyme, N515‐02) were activated just before use with Ca^++^ and Mn^++^ as described in the kit. The cells were collected and incubated in nuclei isolation buffer (5%BSA, 0.1%CA‐630, 1 mm DTT, 1 × Roche Complete Protease Inhibitor in DPBS) on ice for 3 min. A total of 10000 nuclei were washed once and resuspended in 50 µL of wash buffer (20 mm HEPES pH 7.5, 150 mm NaCl, 0.5 mm spermidine). The cell suspension was then mixed with 5 µL of activated ConA beads in PCR tubes and incubated at room temperature for 10 min. Then use a magnet to discard the supernatant. The beads were resuspended in 50 µL of antibody buffer (2 mm EDTA, 3% BSA, 0.05% digitonin, 1 × Roche Complete Protease Inhibitor in wash buffer) pre‐mixed with antibody (1:100) with gentle vortexing, and incubated on a rotator at 4 °C overnight.

Following a quick spin, the bead suspension was discarded by using a magnet to remove the supernatant. Then the beads were resuspended in 50 µL of secondary antibody buffer [0.1% BSA, 1 × Roche Complete Protease Inhibitor in dig‐wash buffer (0.05% digitonin in wash buffer)] pre‐mixed with secondary antibody (1:100). The tubes were placed on a rotator for 1 h at room temperature (10 rpm), the beads were washed three times with 200 µL of dig‐wash buffer. Then the beads in each tube were suspended in 50 µL of dig300‐wash buffer (20 mm HEPES pH 7.5, 300 mm NaCl, 0.5 mm spermidine, 0.01% digitonin) pre‐mixed with pG‐Tn5 (1:100). The tubes were fixed on rotator (10 rpm) and incubated for 1 h at room temperature. After a quick spin, the beads were washed three times with 200 µL of dig300‐wash buffer. 200 µL of tag buffer (10 mm MgCl_2_, 1 × Roche Complete Protease in Dig300‐wash buffer) was added to each well, and the tubes were placed on a rotator at 37 °C for 1 h.

After a quick spin, the beads were then washed once with 200 µL of 10 mm Tris‐HCl and resuspended in 10 µL of 10 mm Tris‐HCl pH 8.0.2 µL of lysis buffer (1.2%SDS, 0.1 mg ml^−1^ Proteinase K in 10 mm Tris‐HCl pH 8.0) was added to each well, and the beads were incubated for 2 h at 65 °C. After a quick spin, 12 µL of 10% Tween‐20 was added to each well and mixed thoroughly. Finally, 25 µL of TruePrep CUT&Tag Amplification Mix (Vazyme, TD612‐01), 0.5 µL of 10 mm MGI‐P5, and 0.5 µL of 10 mm MGI‐P7 primer were added to each well. The PCR program was as follows:72 °C for 5 min; 95 °C for 3 min; 12–15 cycles of 98 °C for 10 s, 60 °C for 5 s; 72 °C for 1 min; and 10 °C hold. All the primers were listed in Table S7 (Supporting Information). Finally, 1.5 × VAHTS DNA Clean Beads were used to purify the library according to the manufacturer's instructions.

### ATAC‐seq

Permeabilized nuclei were counted and diluted in PBST (0.5% Tween‐20 in DPBS) to a concentration of 25000/mL. 2 µL of nuclei suspension was added to 13 µL of tagmentation mix [7.5 µL of 2 × TD buffer (20 mm Tris‐HCl pH7.6, 10 mm MgCl_2_, 20% Dimethylformamide in ddH_2_O), 4.2 µL of PBS, 0.15 µL of 1% digitoxin, 0.15 µL of 10% Tween‐20, 1 µL of TTE (Not diluted with storage buffer)]. Then the tagmentation was carried out at 55 °C for 30 min. Add 200 µL of PBS and centrifuged at 2000 × g for 5 min at 4 °C, and the pellet was directly resuspended in 10 µL RSB buffer. Add 2 µL of lysis buffer (1.2% SDS, 0.1 mg ml^−1^ Proteinase K in 10 mm Tris‐HCl pH 8.0) in each well, and the nuclei were incubated at 55 °C for 30 min. After a quick spin, 12 µL of 10% Tween‐20 was added to each well and mixed thoroughly. Twenty five microliters of 2 × TruePrep CUT&Tag Amplification Mix, 0.5 µL of 10 mm MGI‐P5, and 0.5 µL of 10 mm MGI‐P7 primer were added to each well. The PCR program was as follows: 72 °C for 5 min; 95 °C for 3 min; 11–12 cycles of 98 °C for 10 s, 60 °C for 5 s; 72 °C for 1 min; and 10 °C hold. Finally, 1.5 × VAHTS DNA Clean Beads were used to purify the library according to the manufacturer's instructions

### Processing of RNA‐seq Data

Raw fastq files were processed using Cutadapt [v3.5] to remove adapter sequences (‐a CTGTCTCTTATACACA and ‐A CTGTCTCTTATACACA), with the parameters set to a minimum read length of 20 (‐m 20) and a minimum overlap of 12 bases for adapter matching (‐O 12). Processed fastq files were aligned to mouse reference mm10 using STAR [v 2.7.10a]. Processed fastq files were aligned to the mouse reference mm10 using STAR [v2.7.10a] and converted to BAM files. The aligned BAM files were used with FeatureCounts [v1.6.0] to generate count files, with parameters [‐T 10 ‐t exon ‐g gene_id ‐p].

### Processing of Cut&tag Data

Raw fastq files were processed using Cutadapt [v3.5] to remove adapter sequences (‐a CTGTCTCTTATACACA and ‐A CTGTCTCTTATACACA), with the parameters set to a minimum read length of 20 (‐m 20) and a minimum overlap of 12 bases for adapter matching (‐O 12). Processed fastq files were aligned to mouse reference mm10 using bowtie2 [v2.3.4.3]. Duplicates were removed using Picard [v2.27.5] MarkDuplicates. Aligned reads were converted to BAM and sorted using Samtools [v1.14], with quality filter (“‐q”) set to 2. Reads were then cleaned and sorted by BEDTools [v2.28.0]. In the end, only properly paired mapped reads were kept. Then, H3K4me3 peak calling was performed using MACS3 [v.3.0.0] with parameters [–scale‐to large –keep‐dup all –nomodel –shift 100 –extsize 200]. H3K4me1 peak calling was performed using MACS3 [v.3.0.0] with parameters [–scale‐to large –keep‐dup all –nomodel –shift 100 –extsize 200 –broad].

### Processing of ATAC‐seq Data

Raw fastq files were processed using Cutadapt [v3.5] to remove adapter sequences (‐a CTGTCTCTTATACACA and ‐A CTGTCTCTTATACACA), with the parameters set to a minimum read length of 20 (‐m 20) and a minimum overlap of 12 bases for adapter matching (‐O 12). Processed fastq files were aligned to mouse reference mm10 using bowtie2 [v2.3.4.3]. Duplicates were removed using Picard [v2.27.5] MarkDuplicates. Aligned reads were converted to BAM and sorted using Samtools [v1.14], with quality filter (“‐q”) set to 2. Reads were then cleaned and sorted by BEDTools [v2.28.0]. In the end, only properly paired mapped reads were kept. Then, atac peak calling was performed using MACS3 [v.3.0.0] with parameters [–scale‐to large –keep‐dup all –nomodel –shift 100 –extsize 200].

### Processing of scRNA‐Seq Data

Microwell‐seq data sets were processed as described.^[^ ^45^ ^]^ Reads were aligned to the Mus musculus GRCm38.88 genome using STAR. The DGE data matrices were obtained using the Drop‐seq core computational protocol (available at website http://mccarrolllab.org/dropseq/) with the default parameters. For quality control, cells were filtered out with detection of fewer than 500 transcripts. Cells with a high proportion of transcript counts (>20%) derived from mitochondria‐encoded genes were also excluded. Cells were also corrected for RNA contamination and background‐removed DGE data were constructed as previously described.^[^ ^45^ ^]^ The SCANPY^[^ ^46^ ^]^ python package was used to load the cell‐gene count matrix and perform ln (CPM/100 + 1) normalization. Total UMI was regressed out and each gene was scaled to unit variance with clipping values beyond standard deviation 10. Then, ≈2000 highly variable genes were identified according to their average expression and dispersion and used for principal component analysis and 25 PCs were used for constructing a k‐Nearest‐Neighbor graph. Louvain graph‐clustering method with resolution = 1.3 was applied to identify cell clusters. For visualization, the cell development trajectory was constructed using Force‐directed Layout (FLE) algorithm from Pegasus.^[^ ^47^ ^]^ Marker genes were calculated by the Wilcoxon rank‐sum test using Seurat FindAllMarkers function.^[^ ^48^ ^]^ Cell type and lineage information of each cluster were manually annotated according to the marker genes reported in the previous papers.

For Fluidigm C1 data, the sequenced reads were mapped against the reference Mus musculus GRCm38.88 using STAR. scRNA‐seq expression data, quantified by counts via featureCounts, were analyzed with Seurat. In brief, the Seurat object was generated from digital gene expression matrices. The parameter of “Filtercells” is nGene (2500 to 10000) and mitochondrial percentage (0 to 30%). 2065 variable genes were selected with average expression between 0.01–10 and dispersion > 0.5. Then dimension reduction, clustering and differential gene expression analysis was performed. Cell clusters were annotated using marker genes from literature. Monocle v3^[^ ^49^ ^]^ was used for pseudotime analysis.

### Processing of scATAC‐Seq Data

The original FASTQ files were converted to BAM format first. Then the cell barcodes were extracted and tagged with CB. Three 6 bp barcodes and one 10 bp Tn5 barcode were combined to identify a single cell (a single cell barcode consists of four barcode fragments). Reads with the quality of cell barcodes less than 10 were tagged with XQ and removed afterward. Next, the known barcode sequences involved in the library construction were utilized for cell barcode correction, allowing one mismatch in each barcode fragment. Before alignment, the cell barcodes were added to the query names, the BAM files were converted to the FASTQ files and the 28 bp barcodes at the 5′ end were trimmed simultaneously. BWA (version 0.7.15) was used for read alignment in paired‐end mode. The data were aligned to the mm10 reference genome. Then the aligned BAM files were converted to Fragment files (BED format). The sorted and compressed Fragment files in BED format were required as inputs for subsequent analysis.

To correct the 9 bp gap caused by the transposition reaction, all plus strand insertions were shifted by + 4 bp and all minus strand insertions by – 5 bp using the alignmentSieve function in deepTools.^[^ ^50^ ^]^ Snaptools was again used to convert the aligned and shifted BAM files to SNAP files, which were then converted to Fragment files (BED format). The sorted and compressed Fragment files in BED format were required as inputs for subsequent analysis.^[^ ^51^ ^]^

### Cell Type Comparison Across Different Platforms

To increase the gene coverage in Microwell‐seq data, first data was aggregated from 100 cells in the same cluster to make pseudo‐cells for each cell type. Then MetaNeighbour was used to find the related cell‐type pairs between Fluidigm C1 single cell dataset and Microwell‐seq pseudo cell dataset. MetaNeighbour applied neighbor voting based on the Spearman correlation and the mean AUROC score were obtained from the calculation. Cell type pairs with AUROC score > 0.9 were regarded with strong consistency between two platforms.

### Ancestor States Inference

Waddington‐OT (WOT) was designed to capture the probability distribution of cells in gene‐expression space based on cellular growth and death rates. It inferred how these probability distributions evolve over time with the optimal transport method. First transport maps were computed between pairs of time‐points based on the initial estimate of cell growth rates with growth_iters = 1 (reducing computing time), lambda1 = 1, lambda2 = 50, and epsilon = 0.05. Then, long‐range temporal couplings were inferred based on Markov stochastic process between adjacent time‐points. Then probability vectors for the cell sets in the group 3 on day 35 were generated and the cell set back was pulled through the transport map to infer their ancestors at earlier time points. Cells with a probability more than 0.00025 were considered as parts of the cell cluster trajectory. And then the common ancestor state was inferred and visualized with the contour map. Differentially expressed genes against the rest of clusters were identified using FindMarkers function and gene enrichment analysis was carried out using clsuterProfile^[^ ^52^ ^]^ (p.value<0.01 and q.value<0.05). The ancestor divergence for pairs of trajectories was quantified as 0.5 times the total variation distance between ancestor distributions. Gene trends were computed as the average expression according to this probability distribution.

### Identification of Macrostates and Putative Driving Genes

First, 25% cells were subsampled from the Microwell‐seq data to speed up analysis in CellRank. Initial growth rates were applied for computing transition matrix between adjacent time‐points. Six terminal macrostates in the later days were identified using the combined kernel to initialize a GPCCA estimator and compute a Schur decomposition. Fate probabilities were computed given the terminal states and putative lineage driver genes were inferred by correlating gene expression with fate probabilities.

### Integration and Comparison with Mouse Embryo Development Data

The data of different terminal cells (XEN‐like, Endothelial, Epithelial, Neural cells) were mapped to the reference data embeddings of mouse embryo development^[^ ^15^, ^26^ ^]^ using online update of the scPoli models following the scArches method,^[^ ^53^, ^54^ ^]^ as implemented in the scPoli package, following the manual's tutorial (https://docs.scarches.org/en/latest/).

MetaNeighbour^[^ ^37^ ^]^ was also used to find the related cell‐type pairs between the terminal cell datasets and mouse embryo development cell dataset. Cell type pairs with AUROC score > 0.9 were regarded with strong consistency between two datasets.

### Single‐Cell Trajectory Analysis

In the trajectory inference step, Cellrank (v1.5.1)^[^ ^27^ ^]^ and CytoTRACE^[^ ^55^ ^]^(wrapped in Cellrank) were combined to track the dynamic genetic changes in intermediate cells and computed the pseudotime of XEN‐like, endothelial, epithelial, Neural cells, following the manual's tutorial (https://cellrank.readthedocs.io/en/sytable/beyond_rna_velocity.html). The velocity graph of all intermediate cell was computed using CellRank.^[^ ^27^ ^]^ Gene set variation analysis (GSVA)^[^ ^56^ ^]^ was also performed for the intermediate cell clusters using the R package GSVA.

Moreover, monocle3 (v1.0.0)^[^ ^57^ ^]^ was used to get the inferenced trajectory of XEN‐like, endothelial, epithelial, Neural cells, following the manual's tutorial (https://stuartlab.org/signac/articles/monocle.html).

### Advanced Analyses of scATAC‐Seq

Downstream analyses of scATAC‐seq data were mainly based on R package ArchR.^[^ ^58^ ^]^ The fragment files were input to createArrowFiles function of ArchR (version 1.0.1) to convert chromatin accessibility coordinates into arrow files. The fragments located on chromosome Y and mitochondrial DNA were filtered out by default.

After the generation of all the arrow files, they were loaded into ArchR and combined them into ArchR projects. For quality control, cells with TSS enrichment score greater than 10 and unique fragments greater than 1500 were selected. The potential doublets were filtered out using the filterDoublets function. Tile matrices stored in arrow files divided by 5000‐bp bins were loaded and reduced by addIterativeLSI function. Next, shared nearest neighbor (SNN) in Seurat was introduced for clustering. Data were then embedded using Uniform Manifold Approximation and Projection (UMAP). The chromatin accessibility within a gene body as well as proximally and distally from the TSS was used to infer gene expression via computation of a “Gene Score” using the default method in ArchR. The getMarkerFeatures and getMarkers function (testMethod  =  “wilcoxon”, cutOff  =  “FDR <  =  0.1”) was used to identify the marker regions/genes for each cluster. Finally, the annotations for cell clusters were assigned based on the timepoints and specific markers.

ArchR was also used to analysis pseudotemporal changes in cell states from scATAC‐seq data. Function addTrajectory was used to reconstruct cellular trajectory that approximates the transition from MEFs to intermediate cells. Dynamically accessible regions and enriched motifs along pseudotime were computed by getTrajectory (parameters: useMatrix = “GeneScoreMatrix”) and getTrajectory (parameters: useMatrix = “MotifMatrix”), respectively. Function plotTrajectory was used to exhibit the pseudotime versus the gene score or motif accessibility of the specific genes or TFs, respectively. Integrative analyses was performed to identify positive TF regulators by integration of gene score with motif accessibility across pseudotime. Function correlateTrajectories was used to take two SummarizedExperiment objects retrieved from the getTrajectories and the results were displayed by plotTrajectoryHeatmap.

### Single‐Cell Entropy Analysis

Single‐cell entropy estimation was performed using the methods: CCAT (SCENT v.1.0.2).^[^ ^14^ ^]^ To obtain the best performance, normalization was dependent on the computational methods. For CCAT, it was an approximation of network entropy. CCAT was applied to compute the correlations with the connectome and transcriptome based on the “net13Jun12.m” PPIs. CCAT analysis was performed by using a weighted matrix to leverage all the homology genes between human and other species. The weighted matrix was obtained by converting the gene homology relationship (one‐to‐one, one‐to‐many, many‐to‐one and many‐to‐many) into a binary matrix and normalized it to one human gene. The CCAT evaluates biological systems using physical concepts and reflects the physical properties of biological systems.

Sample Information:

For embryonic development^[^ ^15^ ^]^ (n = 111090 cells), the following cell types were included: ectoderm (n = 18516 cells), endoderm (n = 4303 cells), epiblast (n = 14627 cells), extraembryonic (n = 29861 cells), hypoblast (n = 44 cells), ICM (n = 90 cells), mesoderm (n = 34082 cells), primitive streak (n = 9462 cells), and other (n = 105 cells).

For de‐differentiation^[^ ^3^ ^]^ (n = 251203 cells), the following cell types were analyzed: epithelial (n = 11711 cells), epithelial_clust (n = 3332 cells), iPS (n = 56654 cells), MEFs (n = 4550 cells), MET (n = 31241 cells), neural (n = 4452 cells), phase1 (n = 73205 cells), phase1(D1.5) (n = 1956 cells), phase2 (n = 18227 cells), stromal (n = 38840 cells), and trophoblast (n = 8991 cells).

For regeneration^[^ ^13^ ^]^ (n = 18442 cells), the cell types studied included: basal (n = 1435 cells), epithelial_1 (n = 1964 cells), epithelial_2 (n = 1964 cells), epithelial_preinjury (n = 1269 cells), hematopoietic (n = 889 cells), intermediate_epithelial_1 (n = 3131 cells), intermediate_epithelial_2 (n = 2708 cells), intermediate_epithelial_3 (n = 1636 cells), mesenchymal_1 (n = 1675 cells), mesenchymal_2 (n = 370 cells), mucosal (n = 191 cells), superficial_epithelial_1 (n = 1533 cells), superficial_epithelial_2 (n = 885 cells), and superficial_epithelial_3 (n = 473 cells).

For trans‐differentiation (n = 298915 cells), the following cell types were included: MEFs (n = 12472 cells), C9 (n = 16542 cells), intermediate (n = 179016 cells), epithelial (n = 18712 cells), endothelial (n = 4744 cells), neural (n = 4309 cells), and XEN‐like (n = 26217 cells).

### Comparison of Single‐Cell Transcriptomes Across Differentiation Pathways

In order to find the related cell‐type pairs and obtain driver genes, MetaNeighbour analysis was applied between the data and the data of embryo development,^[^ ^49^ ^]^ de‐differentiation,^[^ ^3^, ^12^ ^]^ and regeneration.^[^ ^13^, ^59^ ^]^ Cell type pairs with AUROC score > 0.9 were regarded with strong consistency between two datasets. Genes that were both highly expressed in the corresponding pair of cell types were extracted by multiplying the normalized gene expression values. Then the clusterProfiler (v4.4)^[^ ^52^ ^]^ package was used to perform GO enrichment analysis for these genes.

## Conflict of Interest

The authors declare no conflict of interest.

## Author Contributions

E.W., L.F., J.W., X.W., and R.W. contributed equally to this work. X.H. conceived the study. X.H. supervised the study. X.H., R.W., X.‐Y.W., J.C., J.‐Q.W., M.J., and D.J. designed and performed experiments. W.E., L.F., X.‐R.W., P.Z., and D.H. data analyses. X.H., W.E., L.F., J.‐J.W., and G.G. performed writing.

## Supporting information



Supporting Information

Supplemental Table 1

Supplemental Table 2

Supplemental Table 3

Supplemental Table 4

Supplemental Table 5

Supplemental Table 6

Supplemental Table 7

Supplemental Table 8

## Data Availability

The data that support the findings of this study are openly available in [Gene Expression Omnibus] at [https://www.ncbi.nlm.nih.gov/geo/], reference number [179357].
