# Supplementary material for: Dynamics of Cell Fate Decisions during Chemically Induced Multi‐Lineage Trans‐Differentiation at Single‐Cell Level
Source: Adv Sci (Weinh). 2025 Mar 7;12(17):2409642. doi: 10.1002/advs.202409642 (PMC12061290; doi:10.1002/advs.202409642)
Supplement: Supplementary file 1 — Supporting Information [file ADVS-12-2409642-s006.docx]

Supporting Information

**Dynamics of Cell Fate Decisions During Chemically Induced Multi-Lineage Trans-Differentiation at Single-Cell Level**

Weigao E, Lijiang Fei1, Jingjing Wang, Xinru Wang, Renying Wang, Xueyi Wang, Peijing Zhang, Jianhui Chen, Junqing Wu, Mengmeng Jiang, Daosheng Huang, Danmei Jia, Guoji Guo* and Xiaoping Han*

W. E, L. Fei, X. Wang, R. Wang, X. Wang, P. Zhang, J. Chen, J. Wu, D. Jia, G. Guo, X. Han

Bone Marrow Transplantation Center of the First Affiliated Hospital, and Center for Stem Cell and Regenerative Medicine, Zhejiang University School of Medicine, Hangzhou, Zhejiang 310000, China.

J. Wang, M. Jiang G. Guo

Liangzhu Laboratory, Zhejiang University, 1369 West Wenyi Road, Hangzhou 311121, China.

D. Huang

School of Medicine, Tsinghua University, 100191 Beijing, China.

Xiaoping Han

Zhejiang Key Laboratory of Multi-omics Precision Diagnosis and Treatment of Liver Diseases, Hangzhou, Zhejiang 310000, China.

*Correspondence: xhan@zju.edu.cn (X.H.); [ggj@zju.edu.cn](mailto:ggj@zju.edu.cn) (G.G.)


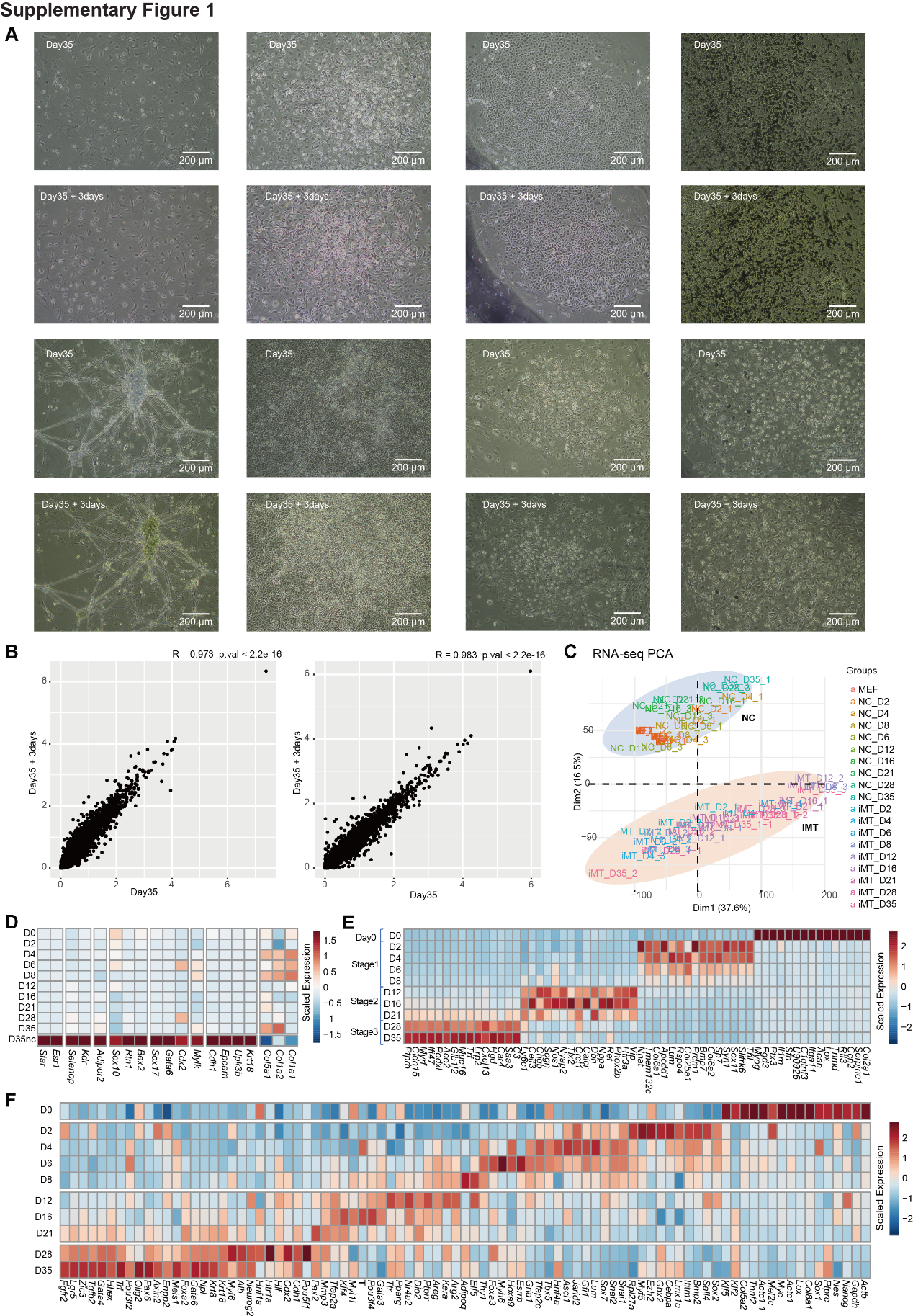


**Figure S1 Morphological and gene expression changes during iMT process.**

1. Pictures of various types of colonies generated via iMT from MEFs. ‘Day35’ represents cells cultured in iMT medium with SMs for 35 days. ‘Day35 + 3days’ represents cells cultured in iMT medium with SMs for 35 days and followed by 3 days without SMs.
2. Scatter plots showing Pearson correlation of gene expression between samples from ‘Day35’ and ‘Day35 + 3days’ without SMs, including two biological replicates.
3. PCA charts demonstrates the distribution of iMT RNA samples and samples that have not been treated with SMs in the PCA space. Percentage stacked chart showing the distribution of samples at all timepoints.
4. A heatmap demonstrating the gene expression of markers for different terminal cell types (rows) across ten time points (columns) of iMT samples. ‘D0’ to ‘D35’ represent cells cultured in iMT medium for 0 to 35 days. 'D35nc' represents cells cultured in MEF medium for 35 days.
5. A heatmap demonstrating the gene expression (rows) of 4 stages (columns) during iMT process.
6. A heatmap demonstrating the gene expression of 89 genes (rows) of 4 stages (columns), selected from Han *et al.*, 2017.


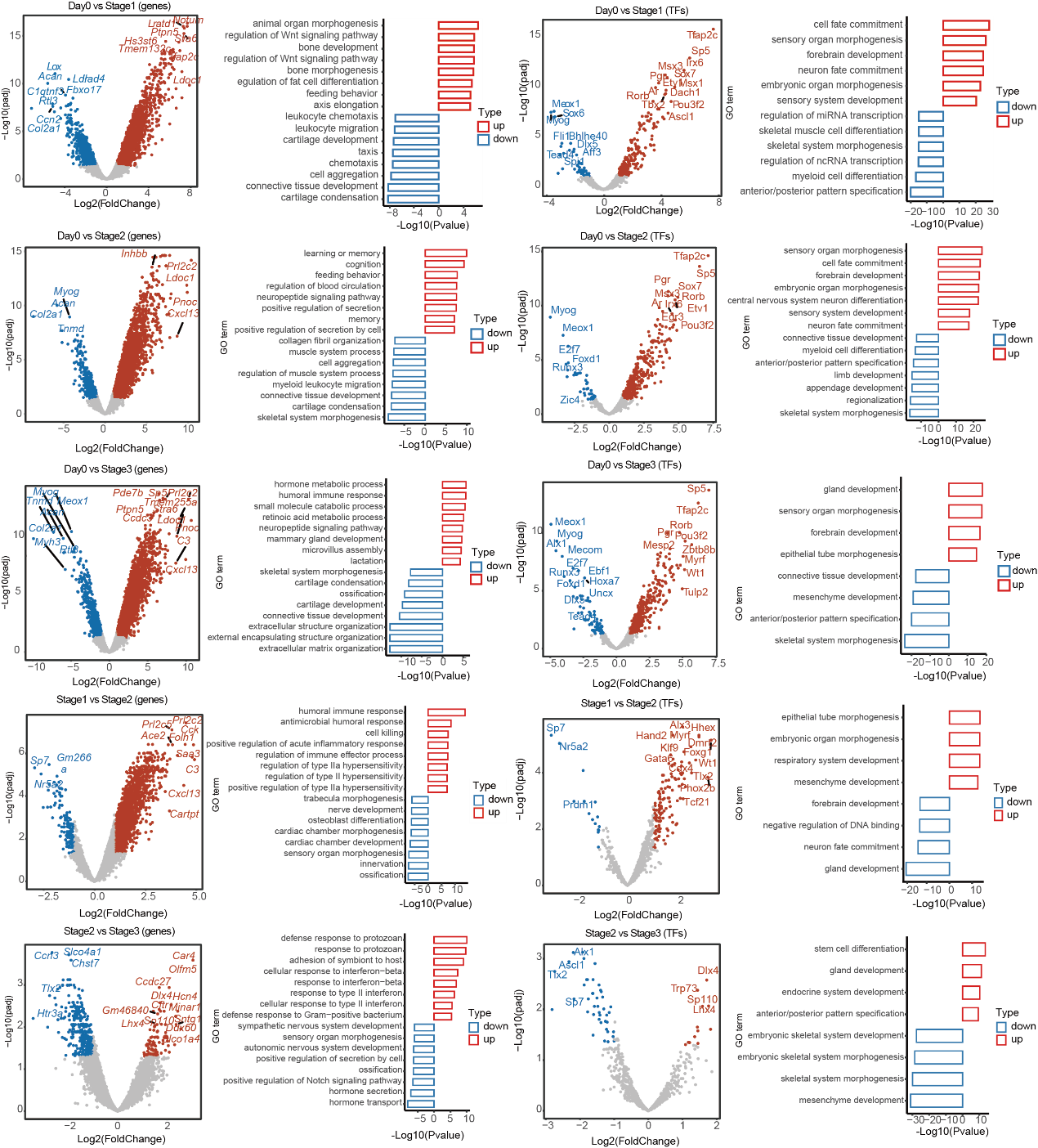


**Figure S2 Gene expression changes over four stages of the iMT process.**

Volcano plots showing the differential TFs and genes between different stages. Barcharts shows the results of the GO BP analysis for the corresponding upregulated and downregulated differential TFs and genes, with the -Log10(Pvalue) of the corresponding downregulated TFs and genes being negative.


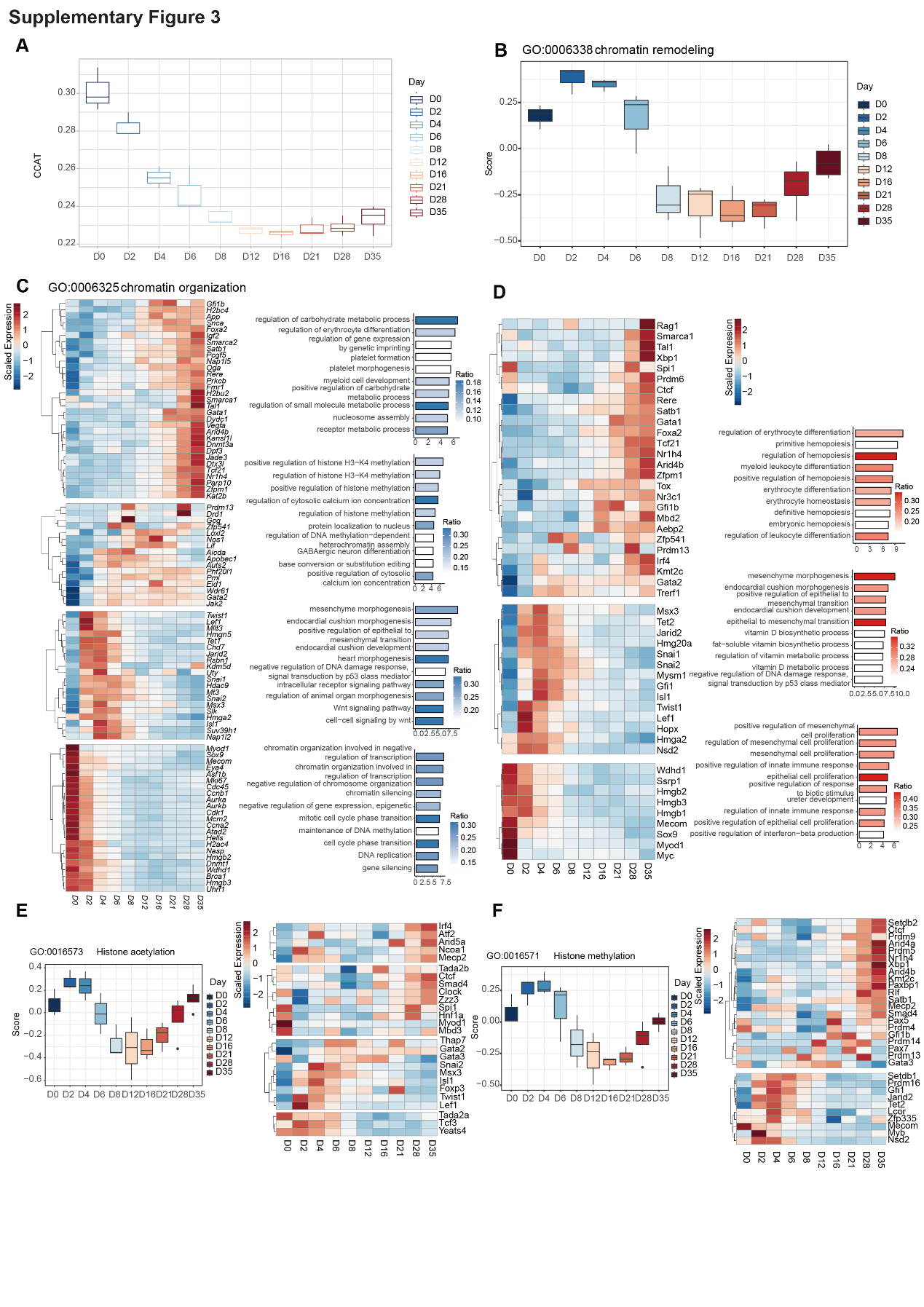


**Figure S3 Chromatin remodeling in iMT process.**

1. A boxplot showing the distribution of the CCAT entropy values from RNA samples (columns) across ten time points (rows).
2. A boxplot showing the GSVA score of GO:0006338 chromatin remodeling from RNA samples (columns) across ten time points(rows).
3. A heatmap displaying the expression of genes in the GO:0006338 chromatin remodeling at ten time points in the iMT RNA (left). Genes have been categorized into four distinct clusters. A barchart shows the results of the GO BP analysis for each gene cluster (right).
4. A heatmap displaying the expression of TFs in the GO:0006338 chromatin remodeling at ten time points in the iMT RNA (left). Genes have been categorized into three distinct clusters. A barchart shows the results of the GO BP analysis for each gene cluster (right).
5. A boxplot showing the GSVA score of GO:0016573 histone acetylation from RNA samples across ten time points (left). A heatmap displaying the expression of TFs in the GO:0016573 histone acetylation at ten time points in the iMT RNA (right).
6. A boxplot showing the GSVA score of GO:0016573 histone methylation from RNA samples across ten time points (left). A heatmap displaying the expression of TFs in the GO:0016573 histone methylation at ten time points in the iMT RNA (right).


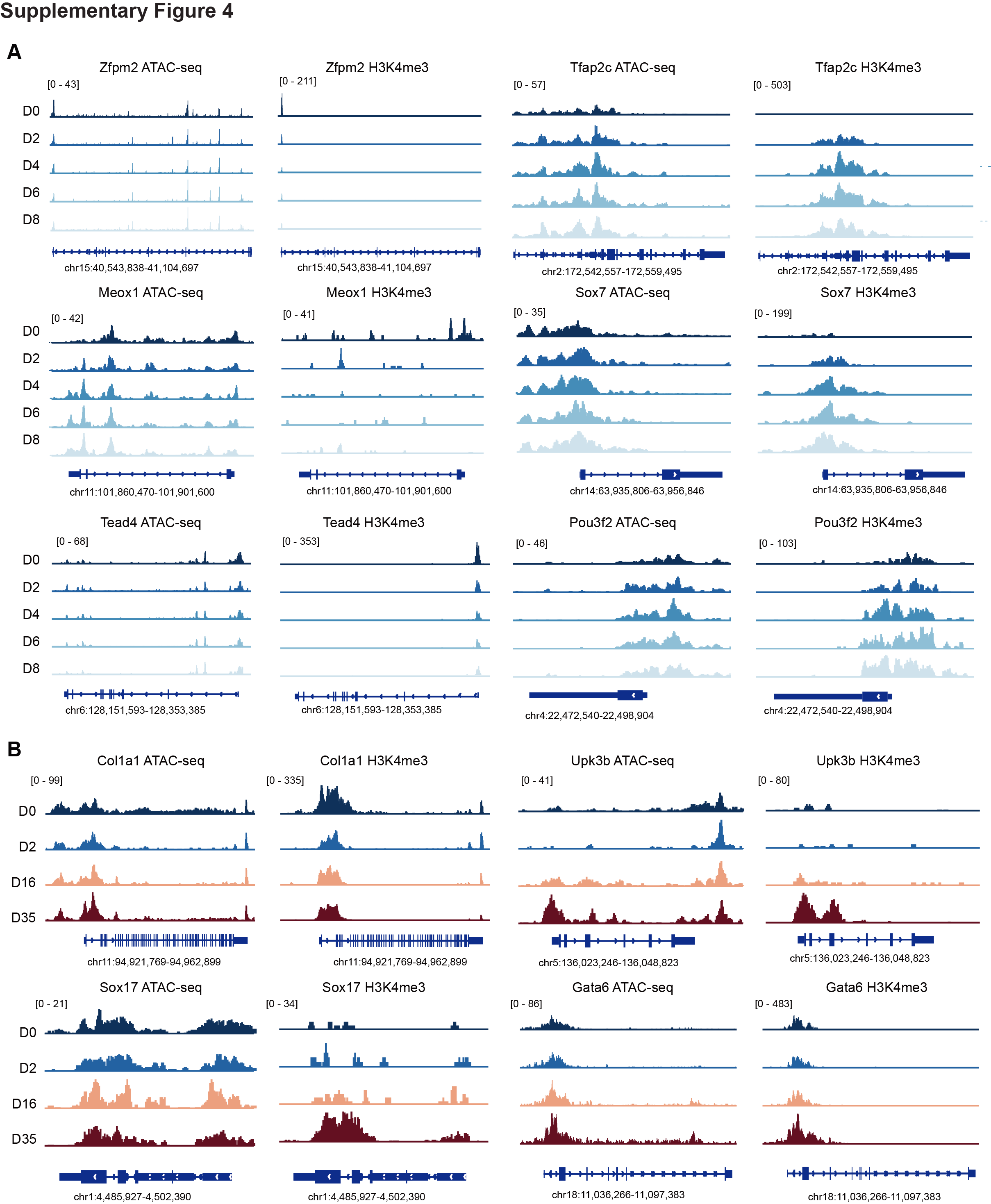


**Figure S4 Normalized sequencing tracks of ATAC and CUT&Tag H3K4me3 peaks.**

1. Normalized sequencing tracks of ATAC and H3K4me3 peaks in D0 and Stage1 (D2, D4, D6, and D8).
2. Normalized sequencing tracks of ATAC and H3K4me3 peaks across different time points (D0, D2, D16, and D35).


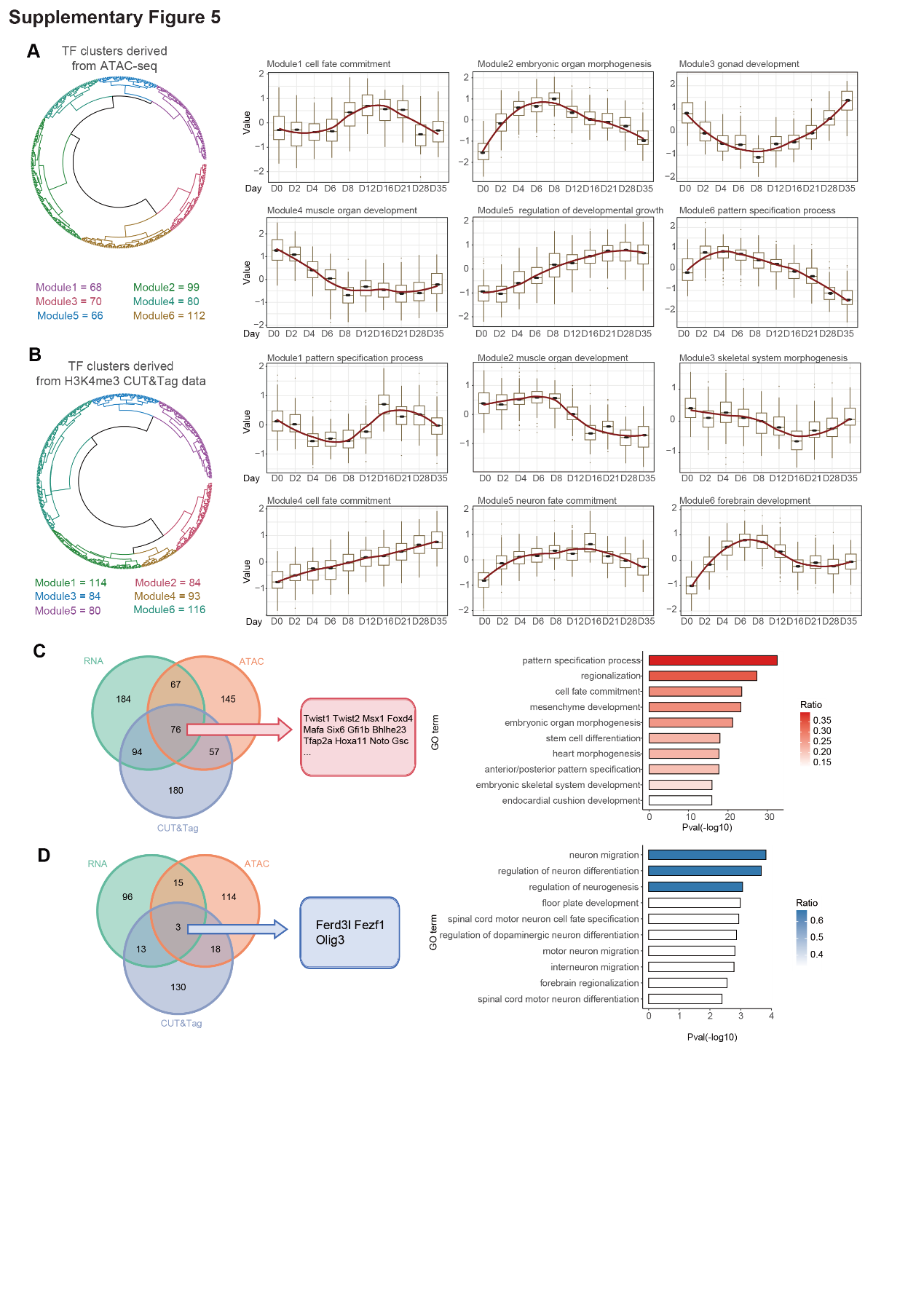


**Figure S5 Multidimensional TF expression pattern clustering analysis highlights commonality.**

1. A circular dendrogram illustrating clustering of TF expression pattern modules over time in ATAC data (left). The boxplots present the average expression of TFs from each cluster at each time point based on the clustering results on the right, along with the most significantly enriched GO terms for the corresponding TF module (right). The red line represents the fitting curve.
2. A circular dendrogram illustrating clustering of TF expression pattern modules over time in CUT&Tag data (left). The boxplots present the average expression of TFs from each cluster at each time point based on the clustering results on the right, along with the most significantly enriched GO terms for the corresponding TF module (right). The red line represents the fitting curve.
3. A venn diagram displaying the common upregulated TFs in the three omics (left). A barchart showing the results of the GO BP analysis for these common upregulated TFs (right).
4. A venn diagram displaying the common downregulated TFs in the three omics (left). A barchart showing the results of the GO BP analysis for these common downregulated TFs (right).


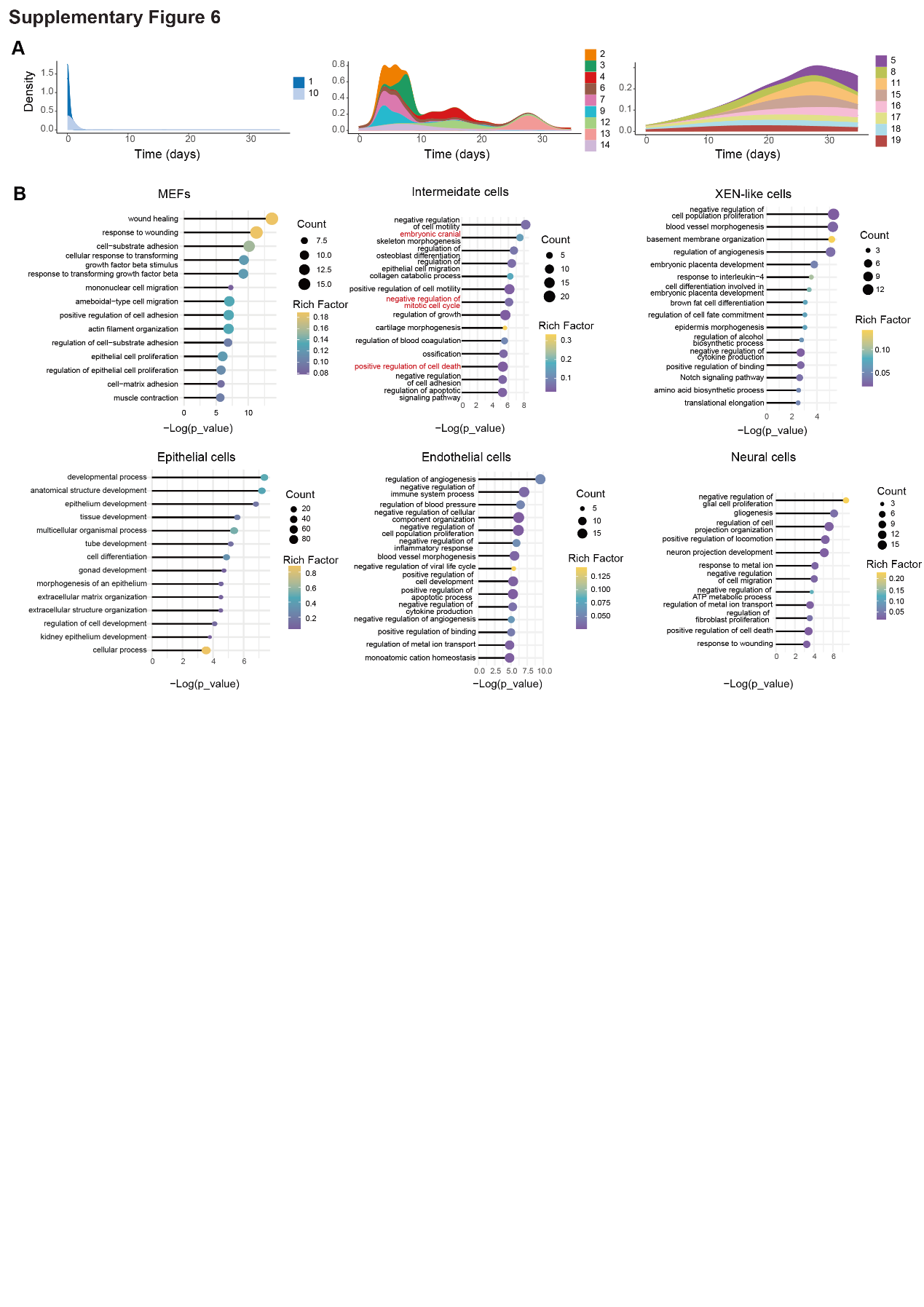


**Figure S6 Characteristics of terminal cells during the iMT process.**

1. Percentage stacked charts showing the distribution of cell density at ten time points, with MEFs on the left, intermediate cells in the middle, and terminal cells along with GM high cells on the right.
2. Lollipop plots showing representative GO terms enriched in marker genes of MEFs, intermediate cells, and four terminal cell types.


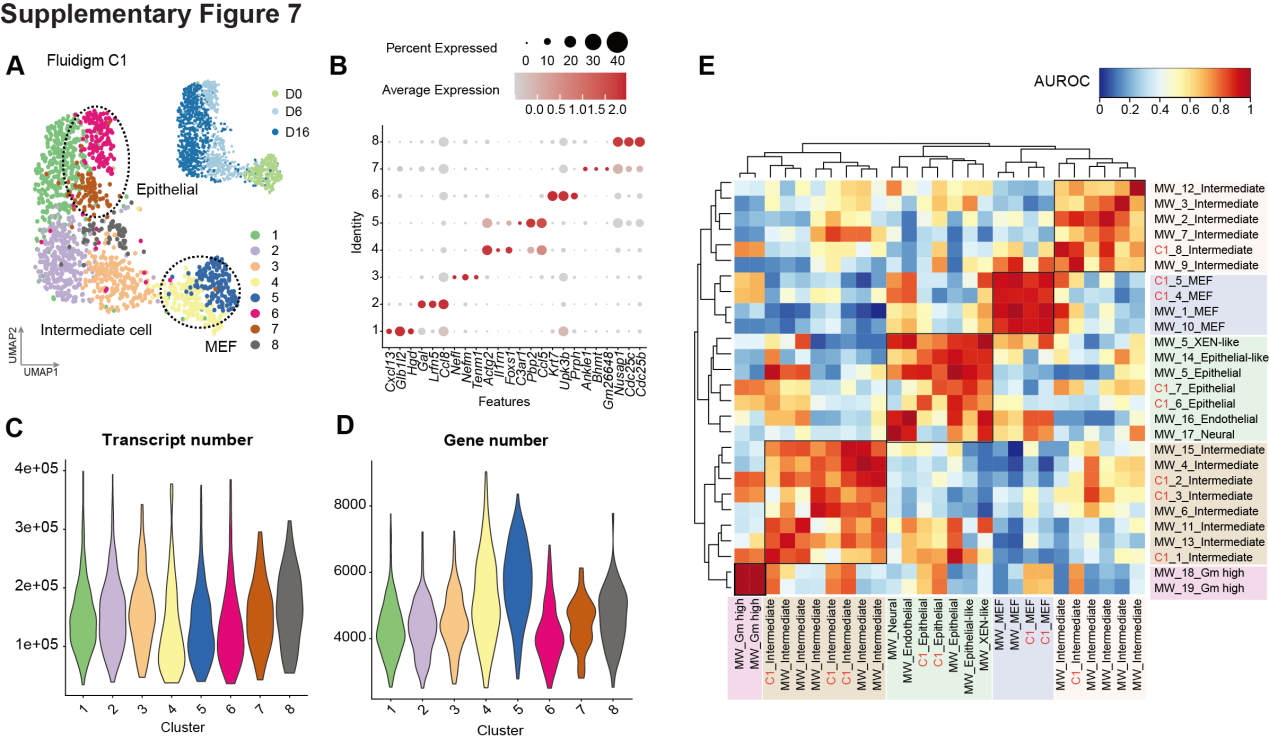


**Figure S7 Mapping cell fates during trans-differentiation using Fluidigm C1.**

1. *t*-SNE visualization of 1,429 single cells from Fluidigm C1, colored by cluster identities and time points.
2. A dotplot showing the expression of the representative genes in cell clusters from Fluidigm C1.

(C-D) Violin plots showing gene numbers (C) and transcript numbers (D) detected in each single cell of each cluster.

(E) A heatmap showing correspondence relationships between clusters from Microwell-seq and Fluidigm C1.


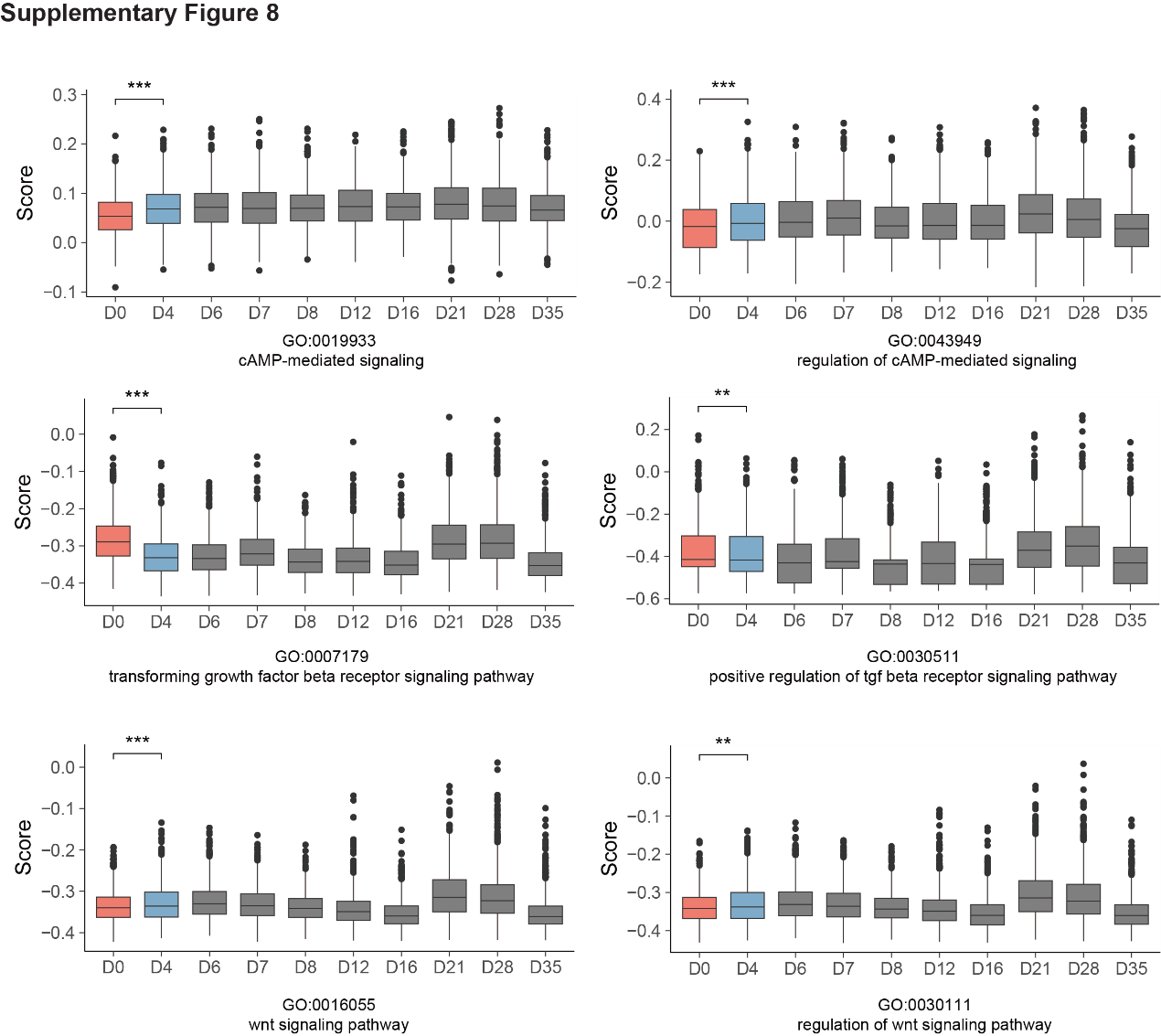


**Figure S8 Significant changes of gene ontology pathway related to SMs during iMT process.**

Boxplots showing the GSVA score of representative GO terms related to SMs during ten time points of iMT process. A t-test was performed for the comparison between the D0 and D4 groups. A single asterisk indicates a significance level of less than 0.05; two asterisks represent a significance level of less than 0.01; and three asterisks signify a significance level of less than 0.001.

**
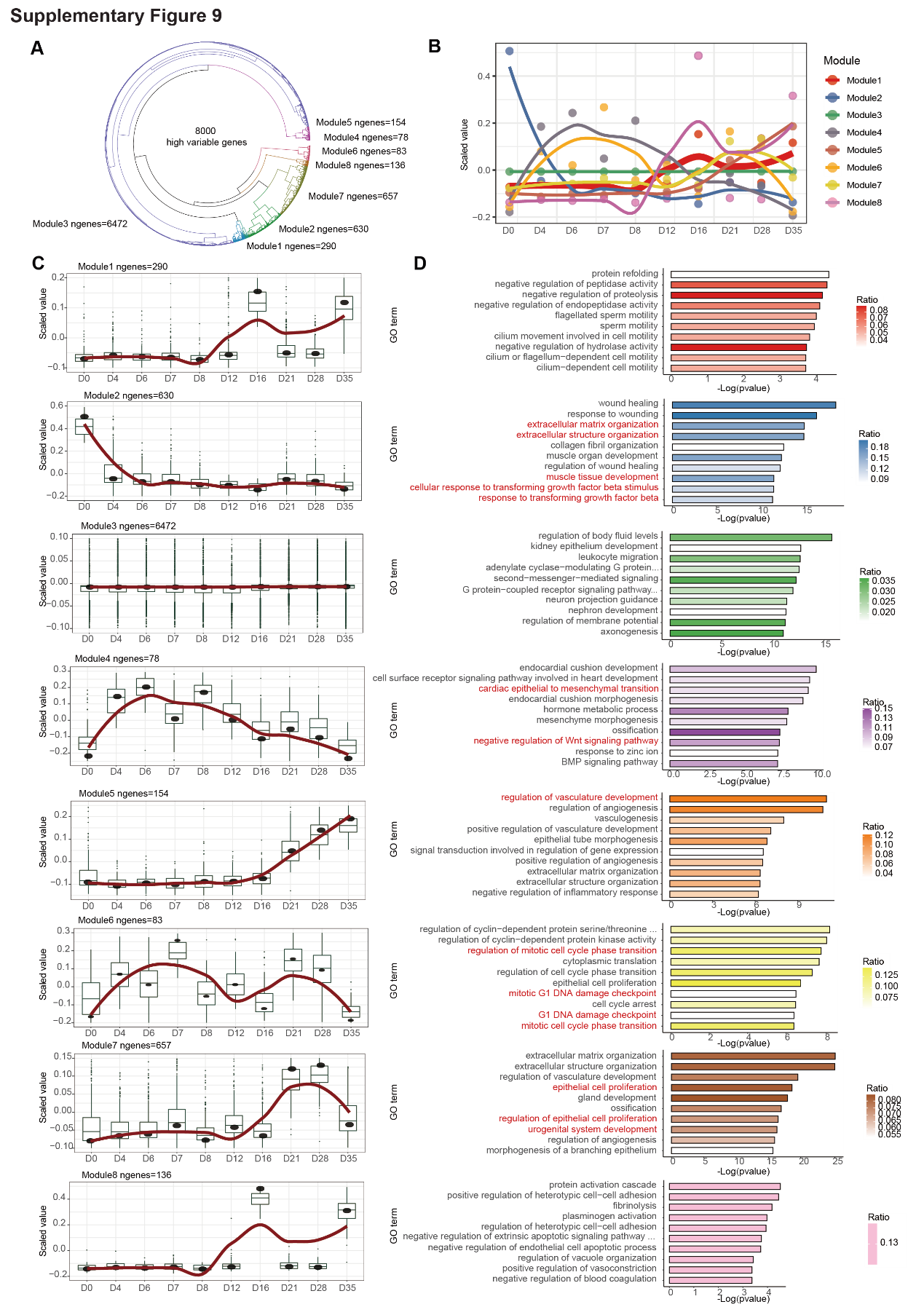
 Figure S9 High variable genes exhibit different patterns during the iMT process.**

1. A circular dendrogram illustrating clustering of 8,000 high variable gene expression pattern modules over time in scRNA-seq data.
2. A dotplot depicting the scaled average expression levels of all genes within each module, with corresponding fitted curves illustrating their changing trends.
3. Boxplots showing the distribution of gene expression levels within each module, with the number of genes indicated. The dots represent the mean values, with corresponding fitted curves showing their changing trends.
4. Barcharts showing the representative GO terms enriched in each gene module.


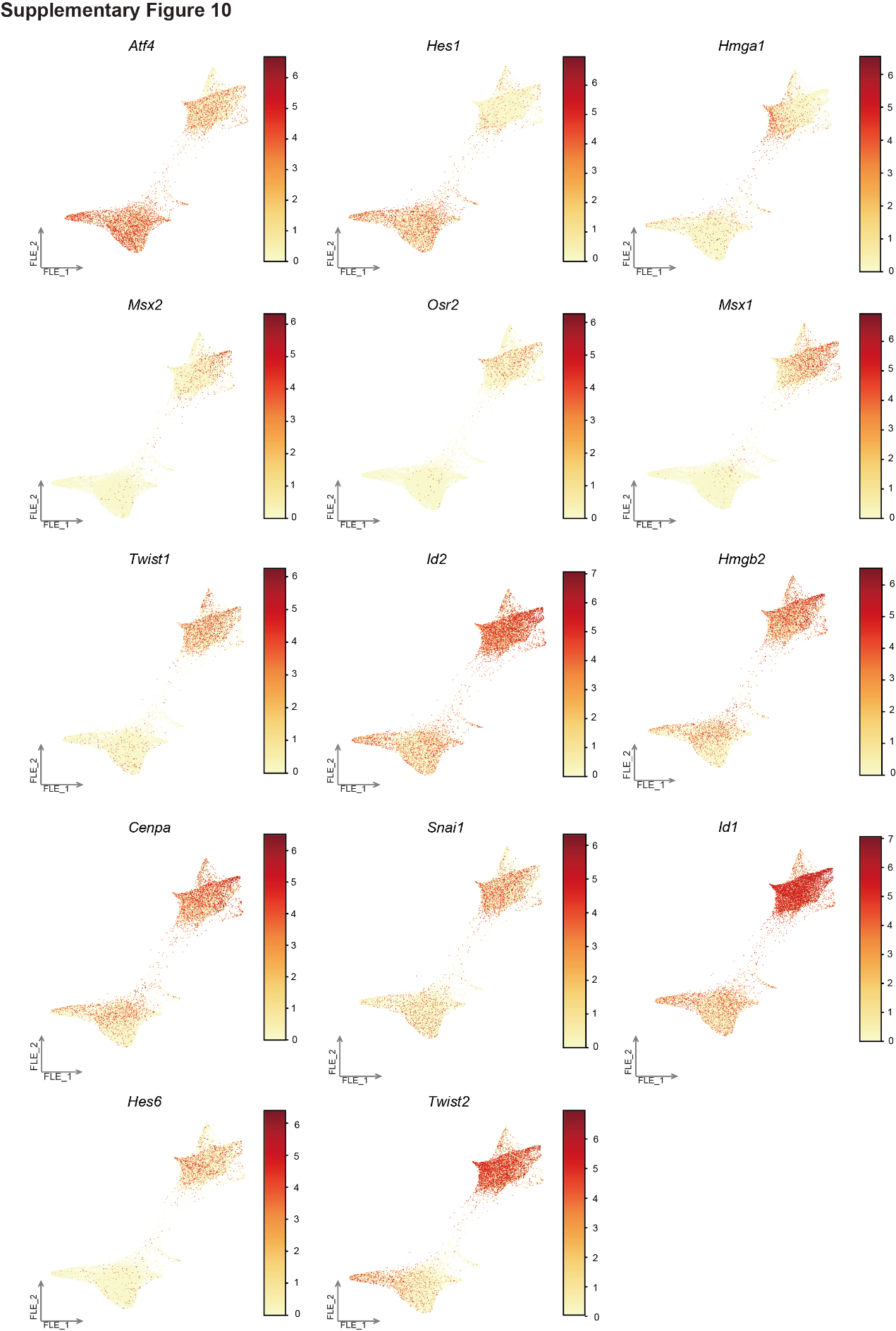


**Figure S10 Expression of the representative genes in MEFs or intermediate cells using Microwell-seq.**

UMAP plots showing the expression of the representative genes in the early and late stages of the trajectory from Microwell-seq.


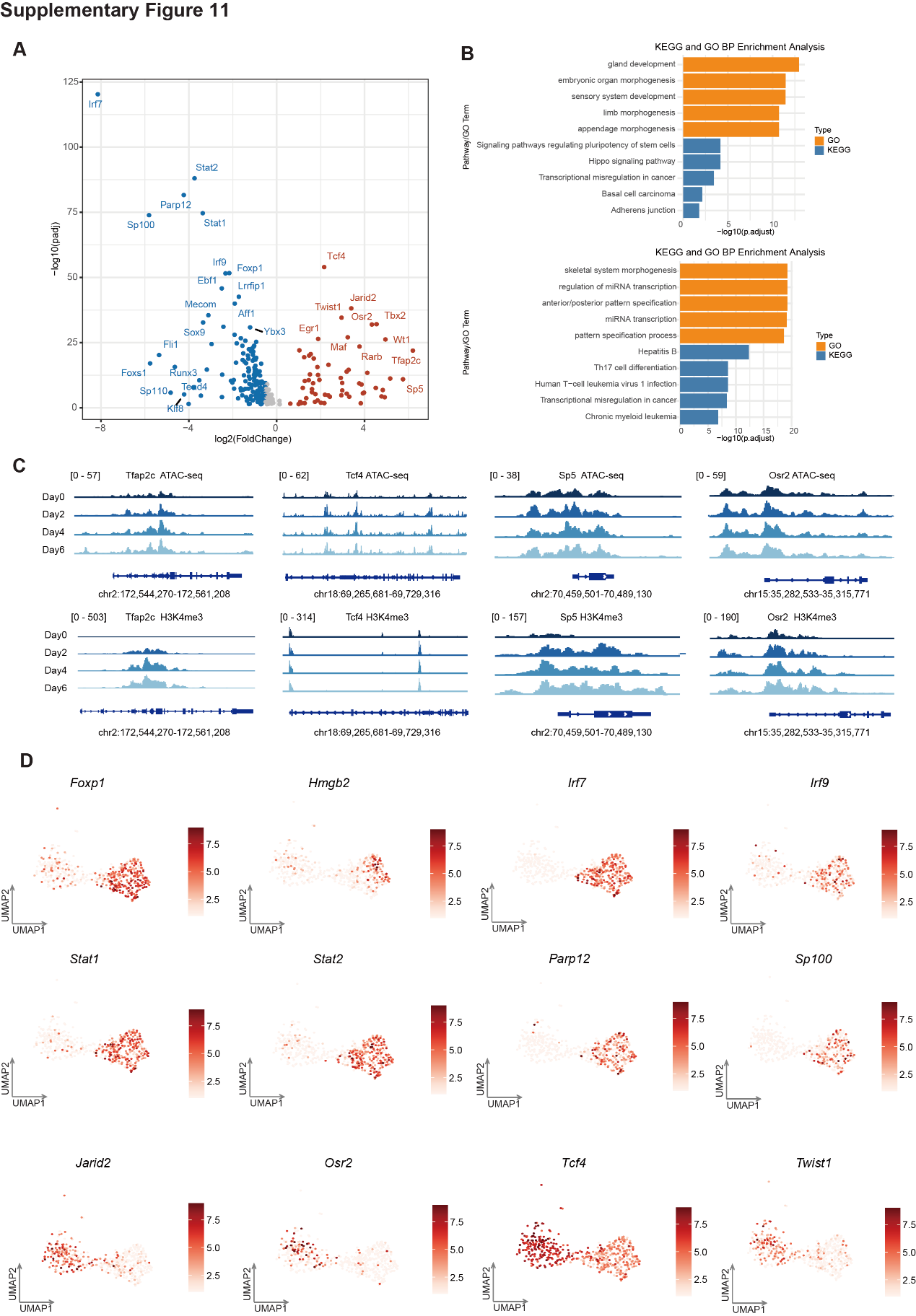


**Figure S11** **Expression of the representative genes in MEFs and intermediate cells using Fluidigm C1.**

(A) A volcano plot showing the differential TFs between Day0 and Day6 of C1 data, where the upregulated TFs in Day6 shown in red and the upregulated TFs in Day0 (downregulated in Day6) shown in blue.

(B) Barcharts shows the results of the GO BP and KEGG analysis for upregulated (top, upregulated in Day6) and downregulated (bottom, upregulated in Day0) TFs, with the -log10(Pvalue) of downregulated genes being negative.

(C) Normalized sequencing tracks of ATAC (top) and H3K4me3 (bottom) peaks across different time points.

(D) UMAP plots showing the expression of the representative genes in the early and late stages of the trajectory from Fluidigm C1 (from Figure 3D).


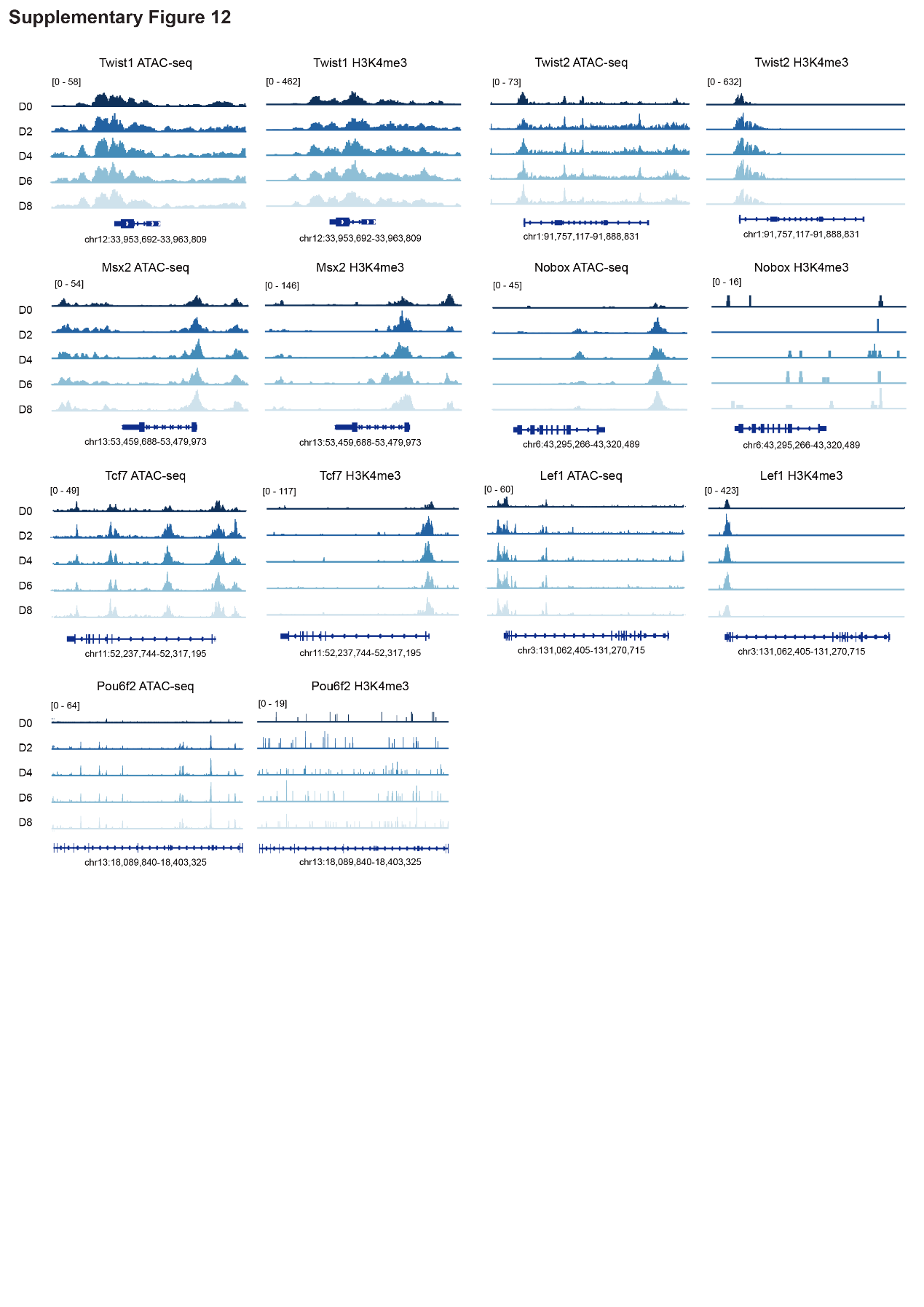


**Figure S12 Normalized sequencing tracks of key TFs peaks in ATAC-seq and CUT&Tag data.**

Normalized sequencing tracks of ATAC-seq and H3K4me3 data key TFs peaks across different time points.

**
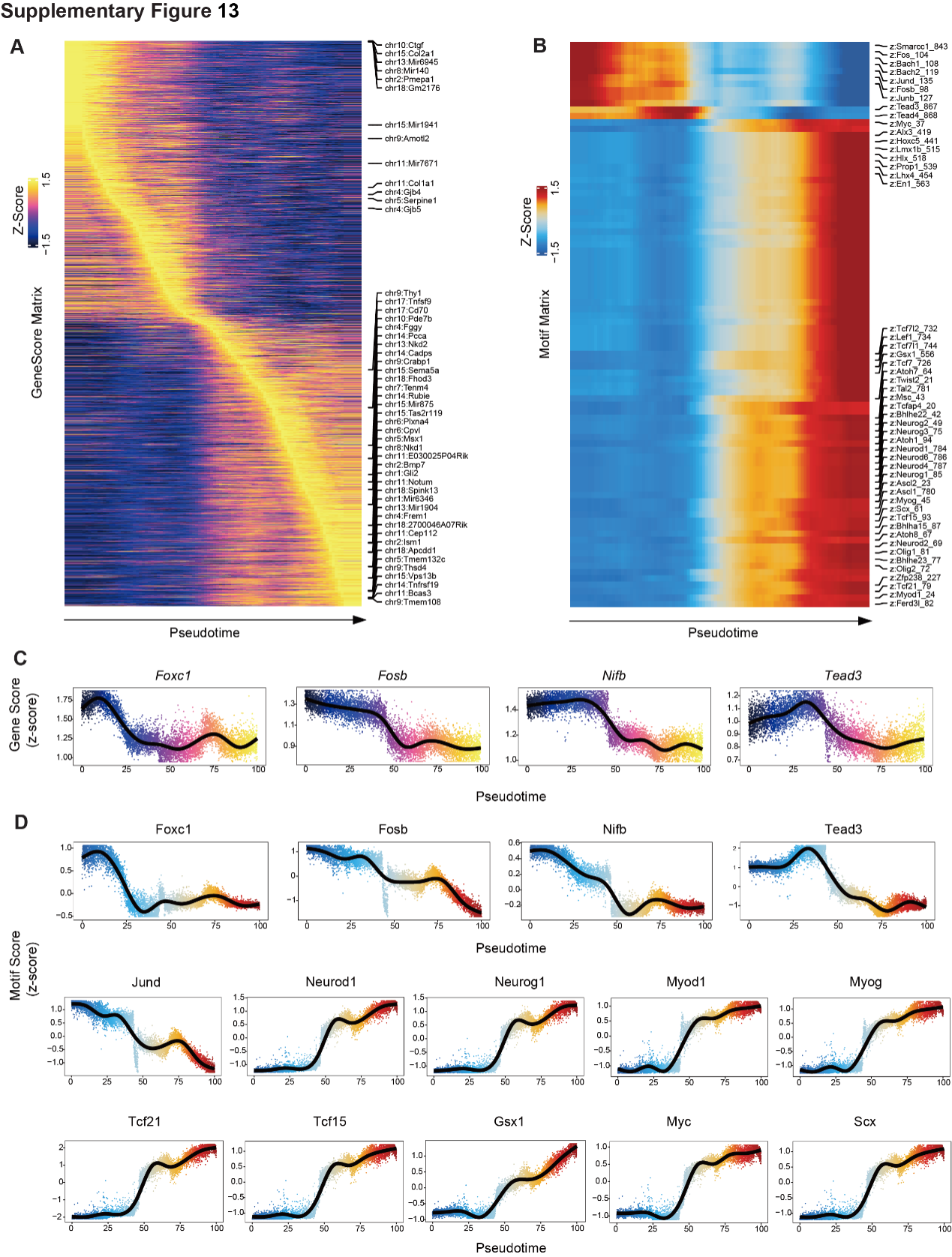
**

**Figure S13 Key genes and TFs that regulate plasticity signatures at the early stages.**

(A-B) A heatmap showing the alternations of enriched TFs based on the gene score matrix (A) and motif enrichment score matrix (B) in pseudotime of the trajectory corresponding to Figure 3i. The 50 genes and TFs with the most significant changes have been marked.

(C) Gene activity of reprehensive down regulated genes along the MEFs to intermediate cells trajectory from scATAC-seq, colored by pseudotime.

(D) Motif deviation of and reprehensive up (top) and down (bottom) regulated TFs along the MEFs to intermediate cells trajectory from scATAC-seq, colored by pseudotime.


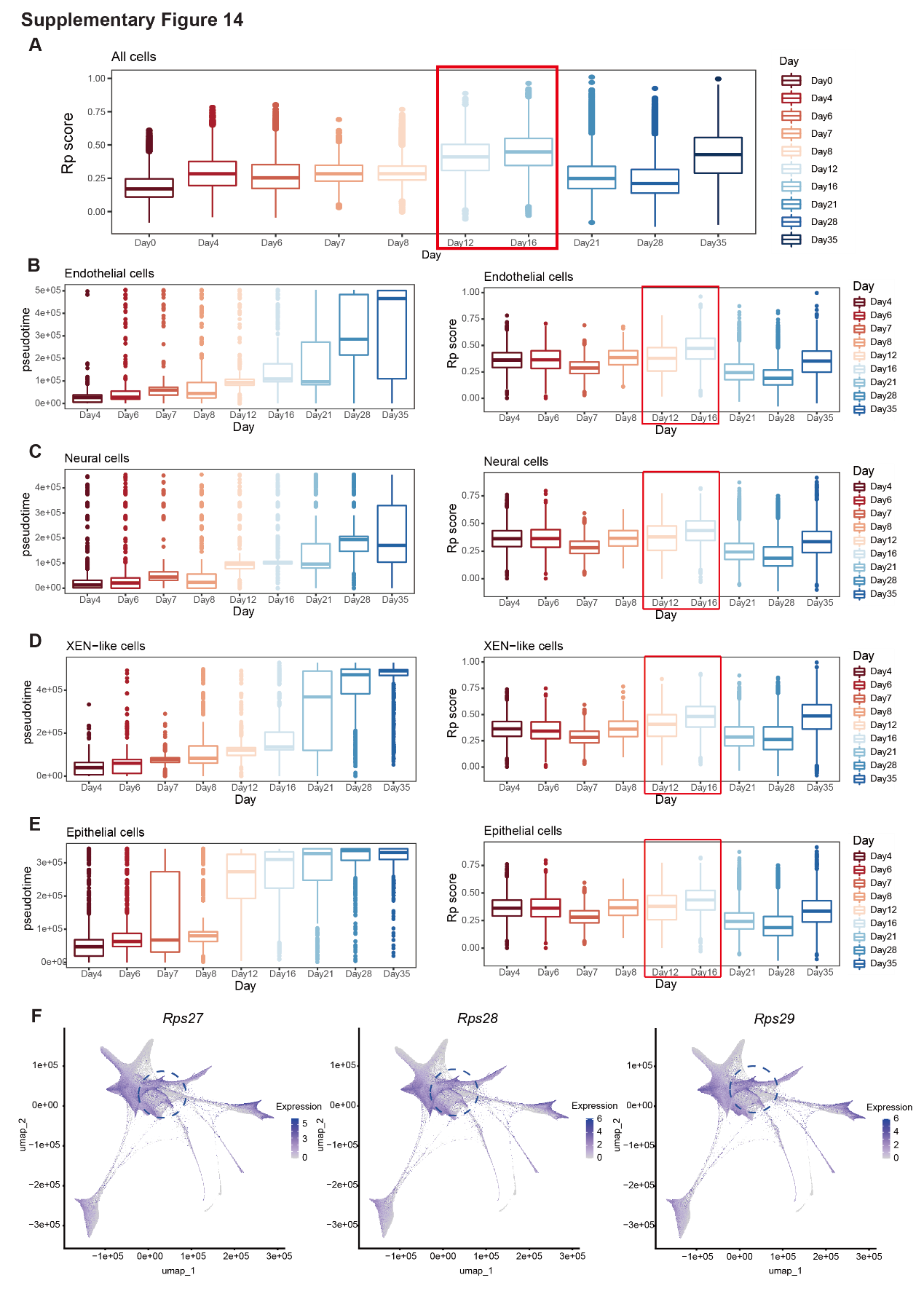


**Figure S14 Intermediate states in the cell trajectory have high ribosomal protein characteristics.**

1. A boxplot depicting the Rp scores (average relative expression of all Rp genes) of all cells at ten time points. The intermediate time points (Day 12 and Day 16) are highlighted in a red box.

(B-E) Boxplots depicting the pseudotime of cells in different cell lineages’ trajectories at nine time points (left) and the corresponding Rp scores of all cells at nine time points (right), including endothelial trajectory (B), neural trajectory (C), XEN-like cell trajectory (D), and epithelial trajectory (E). The intermediate time points are highlighted in a red box.

(F) UMAP plots showing the expression of the representative RP genes in intermediate stages of the trajectory.


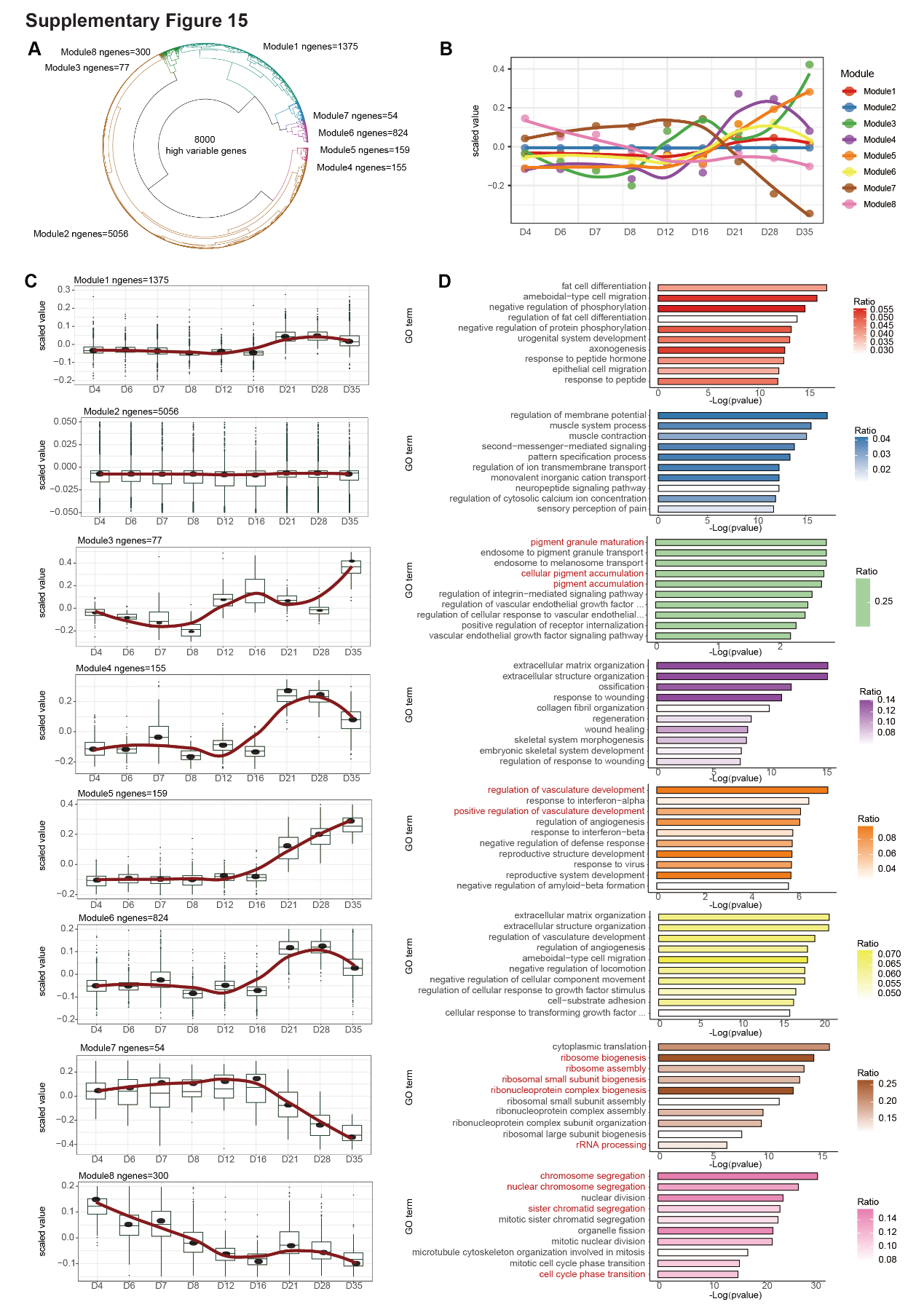


**Figure S15 High variable genes exhibit the same pattern of expression changes in the intermediate cells during the iMT process.**

1. A circular dendrogram illustrating clustering of 8,000 high variable gene expression pattern modules over time in intermediate cells. High variable genes were selected using the FindVariableFeatures function in Seurat with the parameter nFeatures=8000.
2. A dotplot depicting the scaled average expression levels of all genes within each module, with corresponding fitted curves illustrating their changing trends.
3. Boxplots showing the distribution of gene expression levels within each module, with the number of genes indicated. The dots represent the mean values, with corresponding fitted curves showing their changing trends.
4. Barcharts showing the representative GO terms enriched in each gene modules.


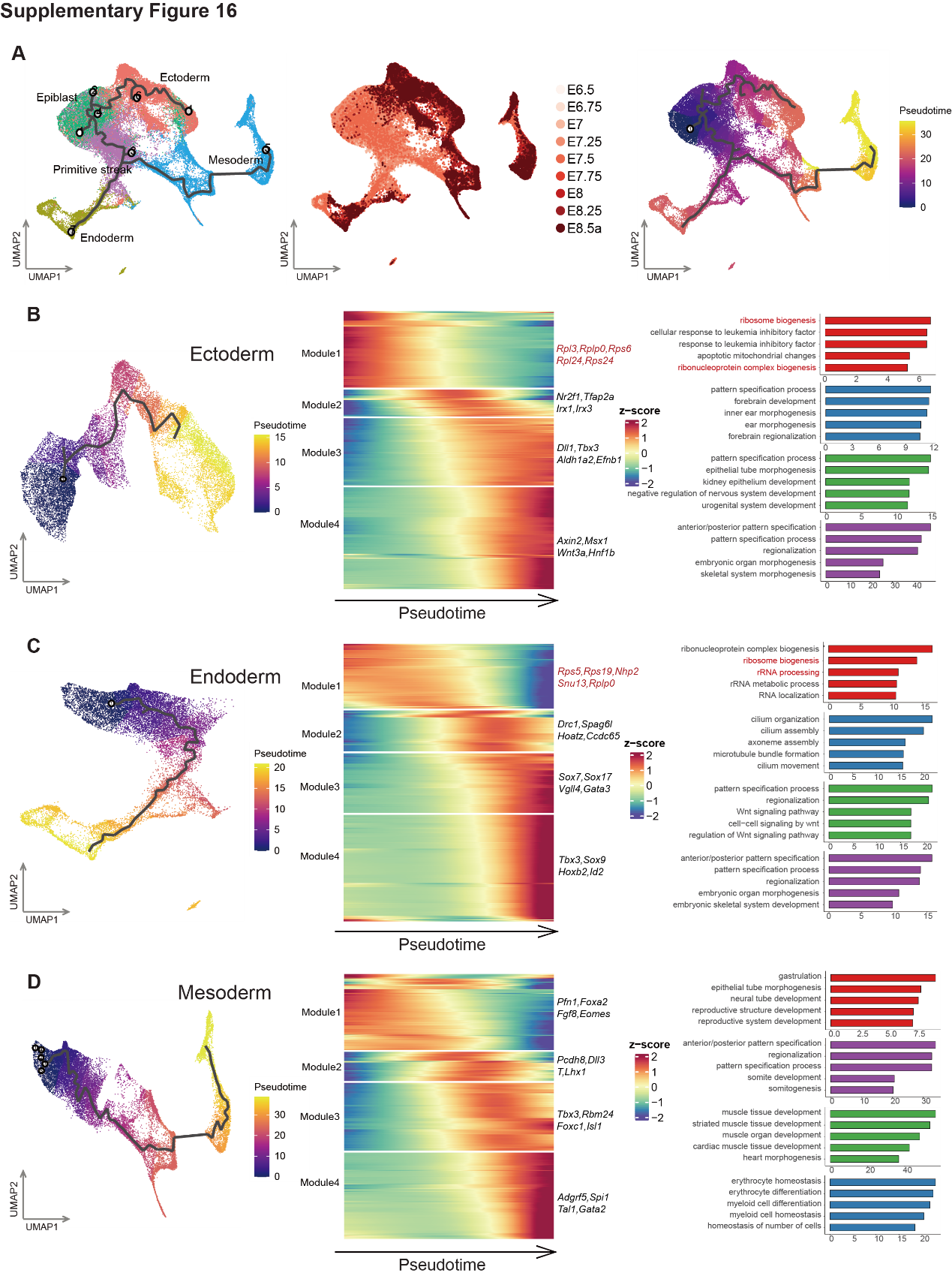


**Figure S16 Reconstruction of embryo development cell trajectory with high Rp gene expression.**

1. UMAP visualization of three main cell trajectories from epiblast to three germ layers from embryo development (Qiu *et al*., 2022), colored by annotation (left), time points(middle) and pseudotime (right).

(B-D) Analyses of cell trajectories from epiblast to ectoderm (B), epiblast to endoderm (C), and epiblast to mesoderm (D), and the corresponding gene modules analyses of these cell trajectories. UMAP visualization of the cell trajectories, colored by pseudotime (left). Heatmaps showing the gene modules at different pseudotime in the corresponding cell trajectories (middle). Barcharts showing representative enriched GO pathways enriched in the corresponding gene modules (right).


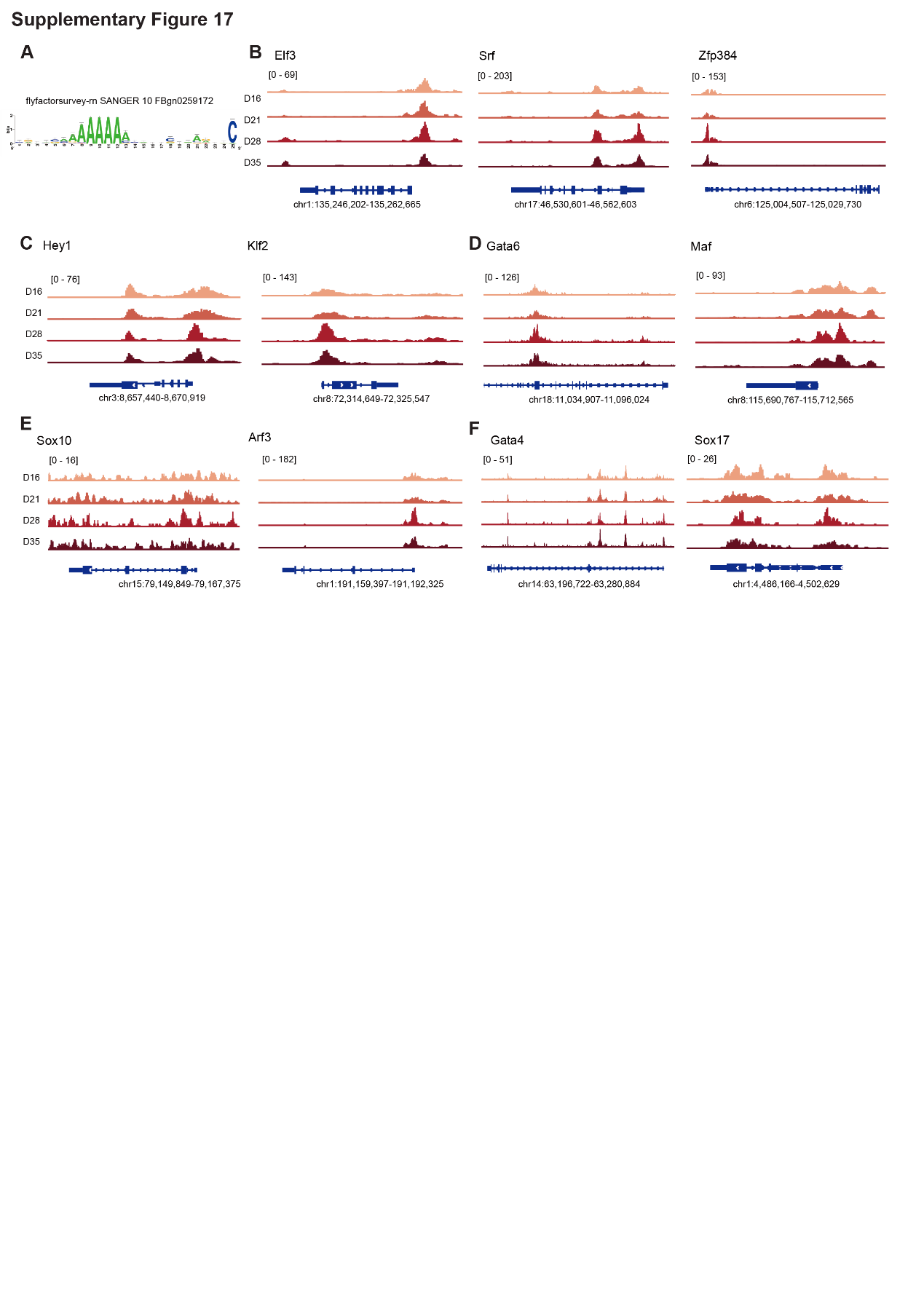


**Figure S17 iMT process and embryonic development go through different cellular states.**

1. Logos of the most significantly enriched motifs identified from the driver TFs of the terminal cell type.

(B) Normalized sequencing tracks of ATAC-seq peaks for the predict TFs of enriched motif across stage2 and stage3 (D16, D21, D28, and D35).

(C-F) Normalized sequencing tracks of ATAC-seq peaks for the driver TFs in endothelial cells (C), epithelial cells (D), neural cells (E), and XEN-like cells (F) across stage2 and stage3 (D16, D21, D28, and D35).


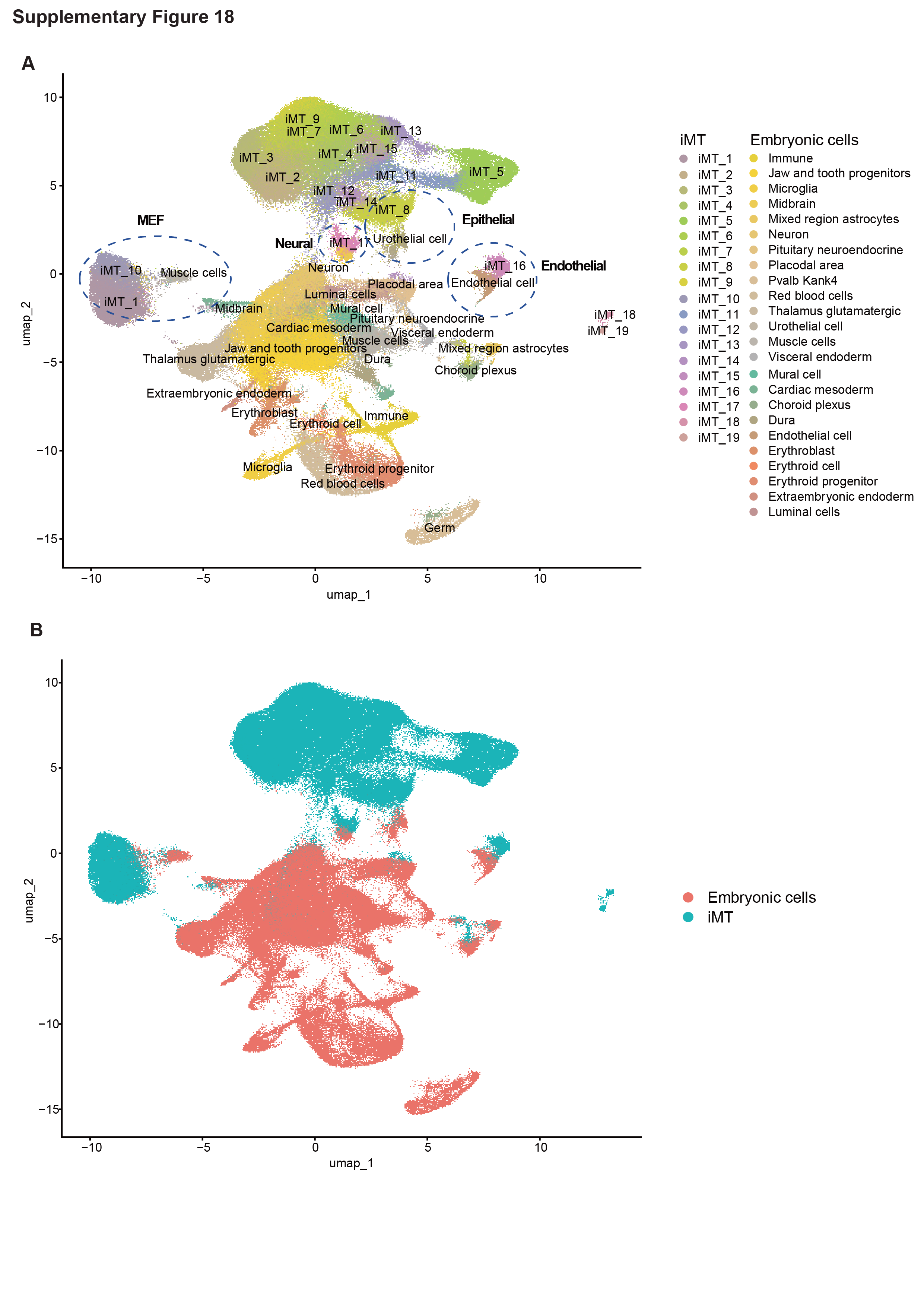


**Figure S18** **iMT process and embryonic development go through different cellular states.**

(A-B) UMAP visualization of the integration of iMT data and embryo development data (Fei *et al*., 2022), colored by cell-type annotation (A) and data resource (B).


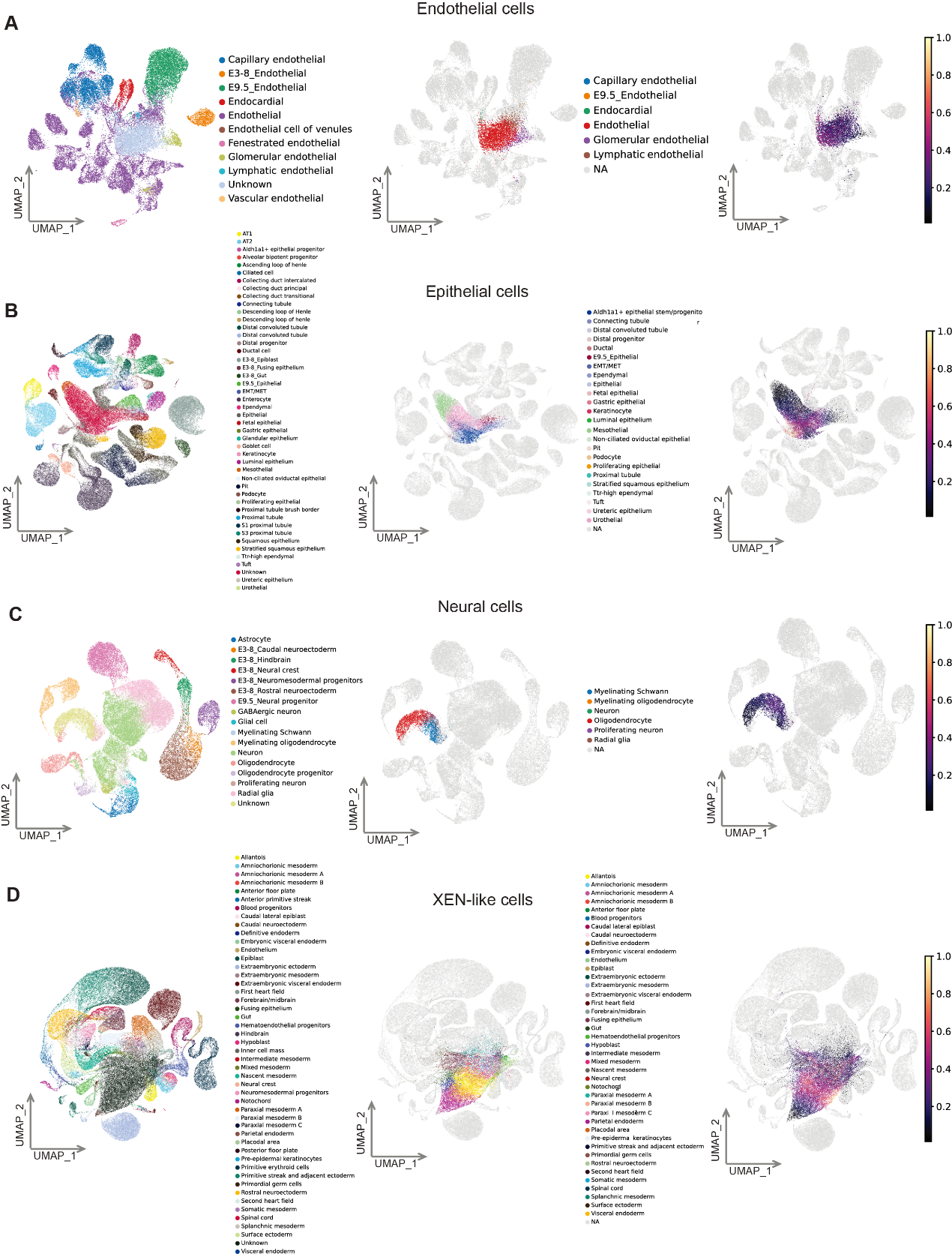


**Figure S19 Comparison cell types between iMT terminal macrostates and mouse embryonic cell.**

(A-D) UMAP visualization of the integration of different cell lineages in our work and the corresponding lineage cells in mouse embryonic development reference (Qiu *et al*., 2022; Fei *et al*., 2022), with a total of 4,744 cells in endothelial (A), 18,712 cells in epithelial (B), 4,309 cells in neural (C), and 26,217 cells in XEN-like (D). UMAP visualizations were colored by original cell-type annotation (left), predicted cell-type annotation (middle), and prediction uncertainty (right), respectively. The UMAP and clustering were calculated using the supervised scPoli model.


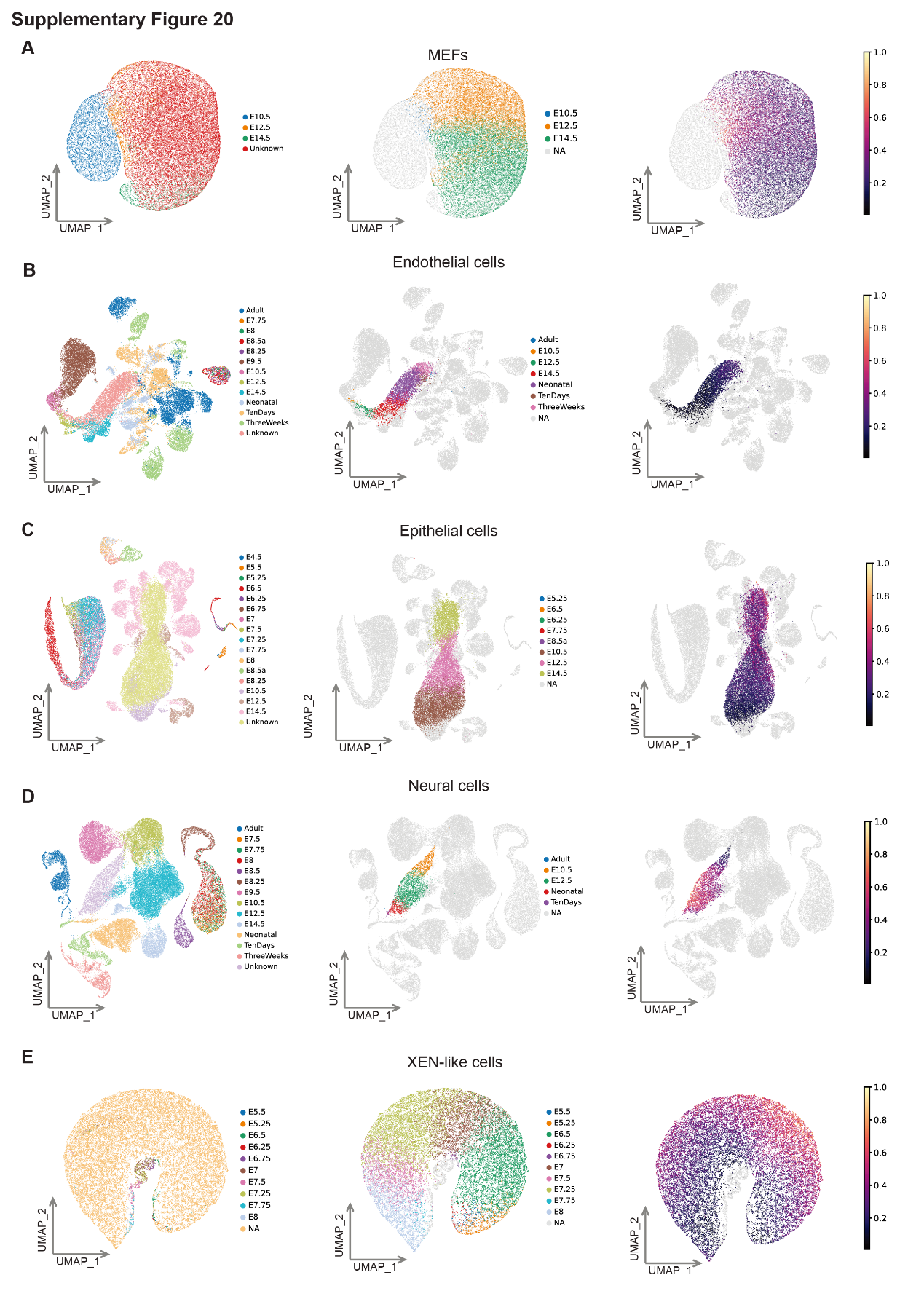


**Figure S20 Comparison time points between iMT terminal macrostates and mouse embryonic cell.**

UMAP visualization of the integration of different cell lineages in our work and the corresponding lineage cells in mouse embryonic development reference (Qiu *et al*., 2022; Fei *et al*., 2022), with a total of 47,169 cells in MEFs (A), 4,744 cells in endothelial (B), 18,712 cells in epithelial (C), 4,309 cells in neural (D), and 26,217 cells in XEN-like (E). UMAP visualizations were colored by original timepoint annotation (left), predicted timepoint annotation (middle), and prediction uncertainty (right), respectively. The UMAP and clustering were calculated using the supervised scPoli model.

**
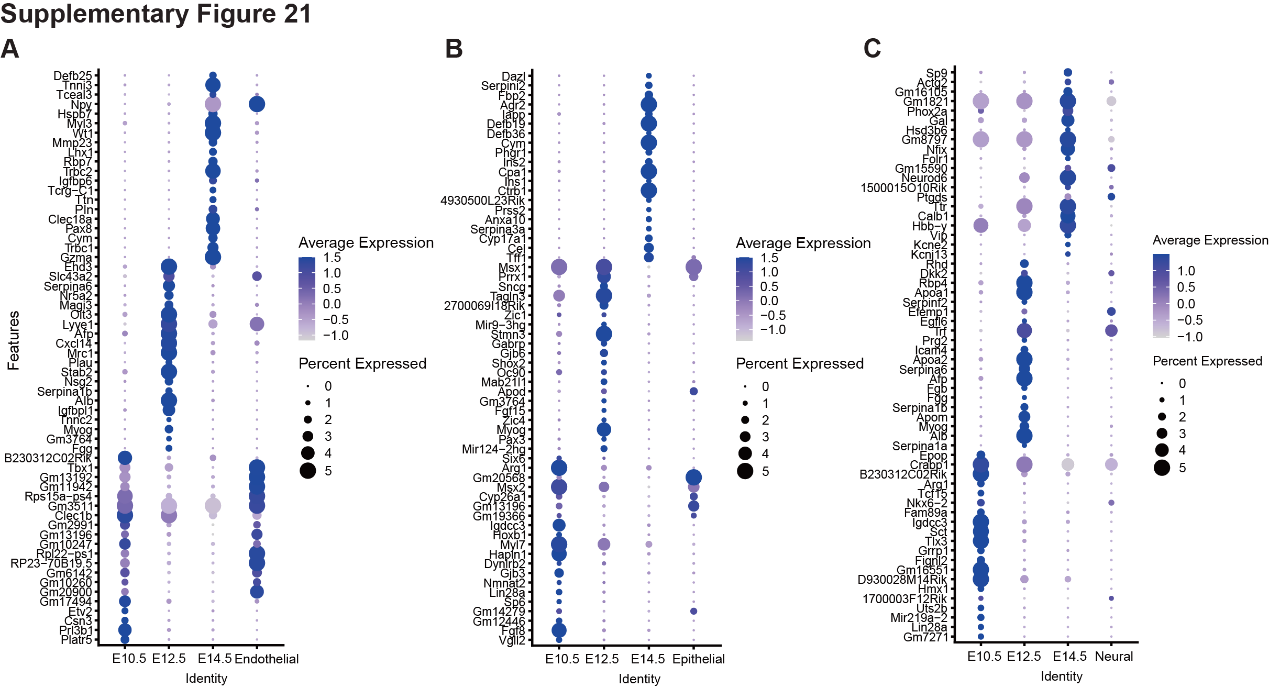
**

**Figure S21** **Scaled average expression levels of top expressed genes across different time points in iMT data and embryonic endothelial, epithelial, and neural cells.**

(A-C) Dotplots depicting the scaled average expression levels of top expressed genes of different cell types in iMT data (this work) and embryo data at different time points, including endothelial cells (A), epithelial cells (B), and neural cells (C).

**
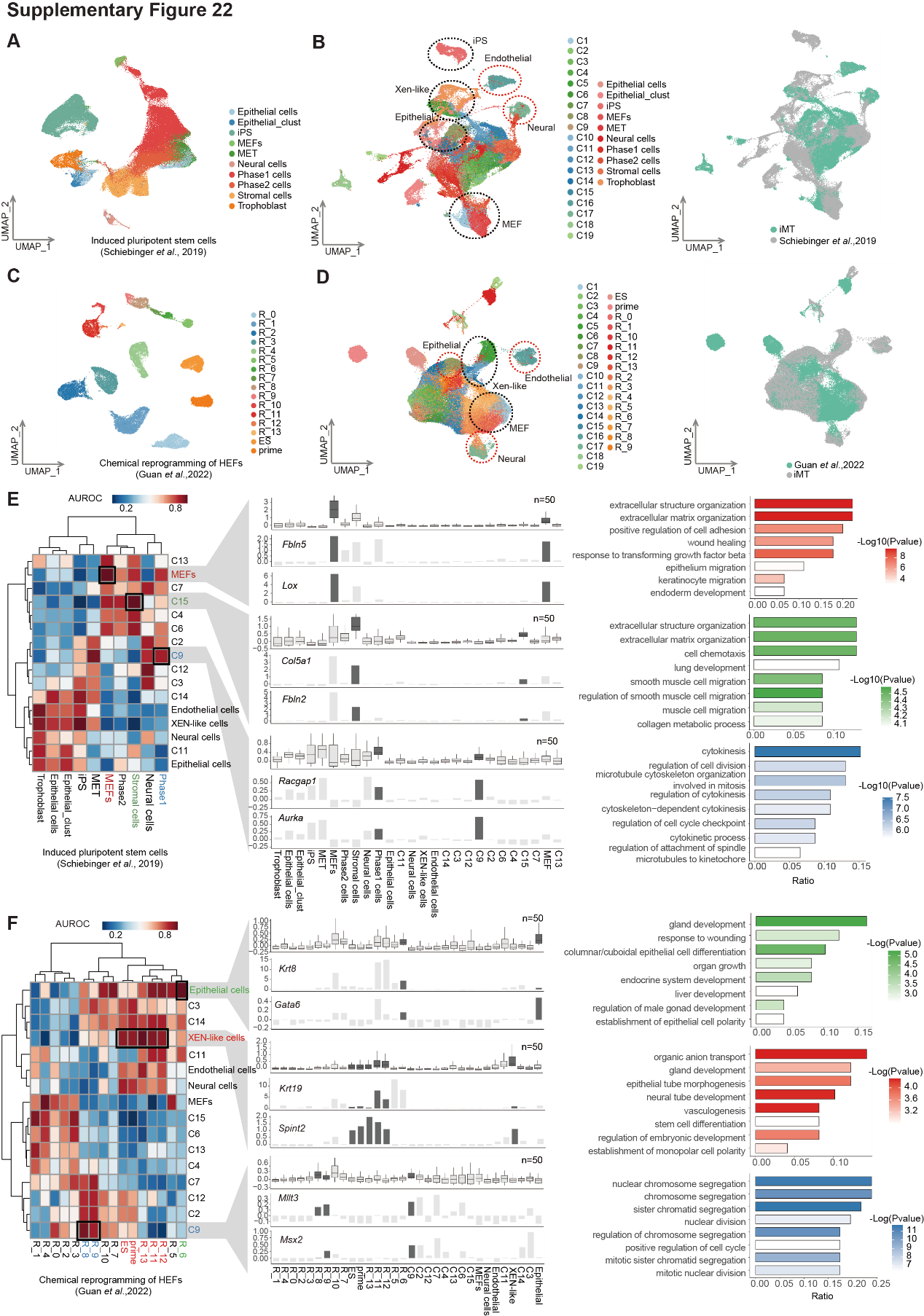
**

**Figure S22** **Comparison between iMT and reprogramming process.**

1. A UMAP visualization of de-differentiation data (Guan *et al*., 2022), colored by cell-type annotation.
2. UMAP visualization of the integration of iMT data (this work) and de-differentiation data (Guan *et al*., 2022), colored by cell-type annotation (left) and data resource (right), respectively.
3. A UMAP visualization of de-differentiation data (Schiebinger *et al*., 2019), colored by cell-type annotation.
4. UMAP visualization of the integration of iMT data (this work) and de-differentiation data (Schiebinger *et al*., 2019), colored by cell-type annotation (left) and data resource (right), respectively.

(E-F) The correlation analysis between cell types of iMT process (this work, column) and two de-differentiation data (row), induced pluripotent stem cell (Schiebinger *et al*., 2019) (E) and Chemical reprogramming of HEF (Guan *et al*., 2019, left) (F). Heatmaps showing the cell type correlation between cell types of iMT process (this work) and de-differentiation data (left). Boxplots showing the average expression of top driver genes promoting specific similarities between cell types in the two process (middle top). Barcharts showing the expression levels of two presentative genes (middle bottom). Representative GO terms enriched in the co-expressed driver genes (right).


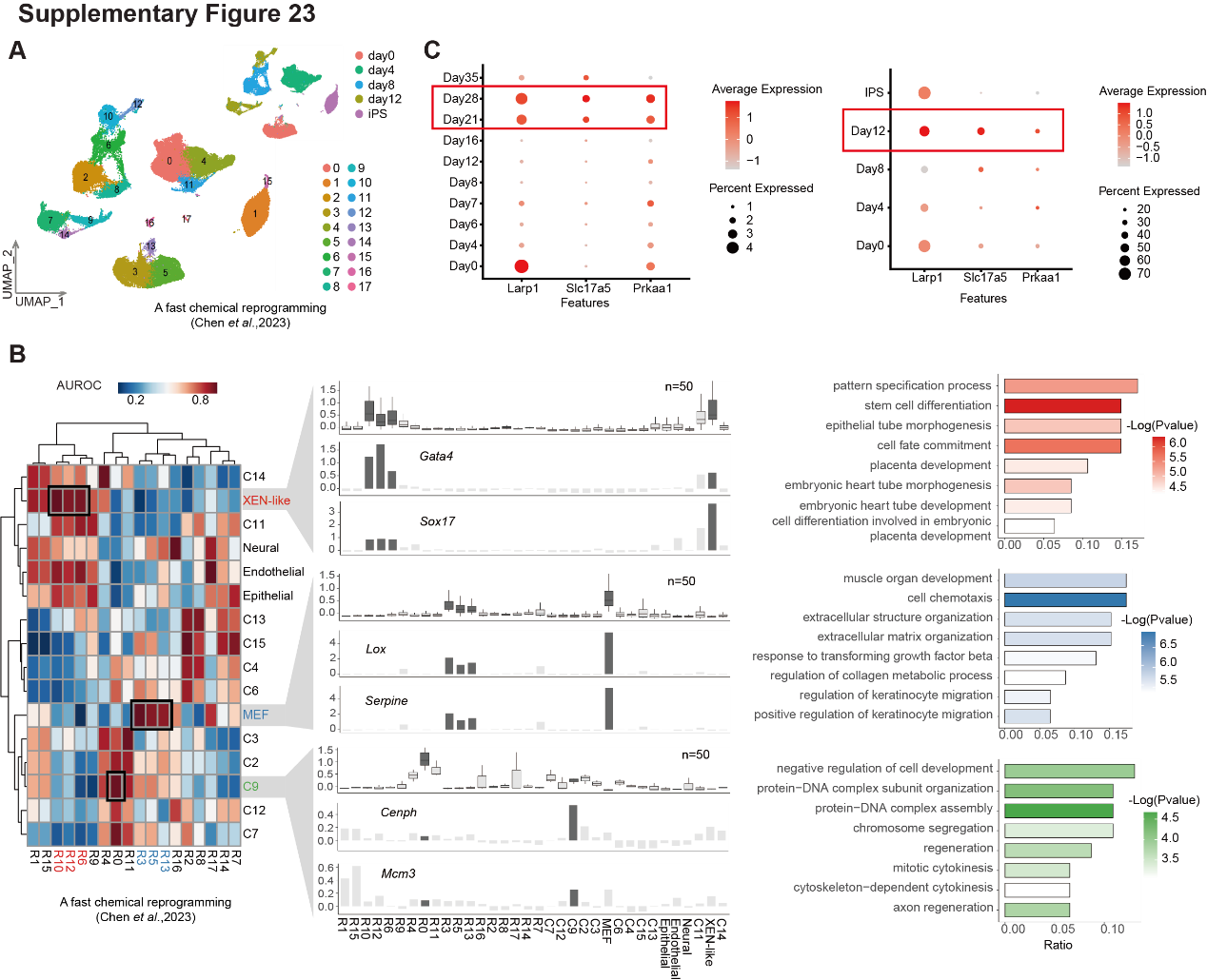


**Figure S23 Comparison between iMT and fast chemical reprogramming.**

1. UMAP visualization of rapid reprogramming data (Chen *et al.*, 2023), colored by clusters and time points.
2. A heatmap showing the cell type correlation between cell types from iMT process (this work) and regeneration process (Chen *et al.*, 2023, left). Boxplots showing the average expression of top driver genes promoting specific similarities between cell types in the two process (middle top). Barcharts showing the expression levels of two presentative genes (middle bottom). Representative GO terms enriched in the co-expressed driver genes (right).
3. Dotplots showing the scaled average expression levels of Diapause-like state marker genes. The size of the points represents the gene expression proportion, and the color represents the relative expression level.


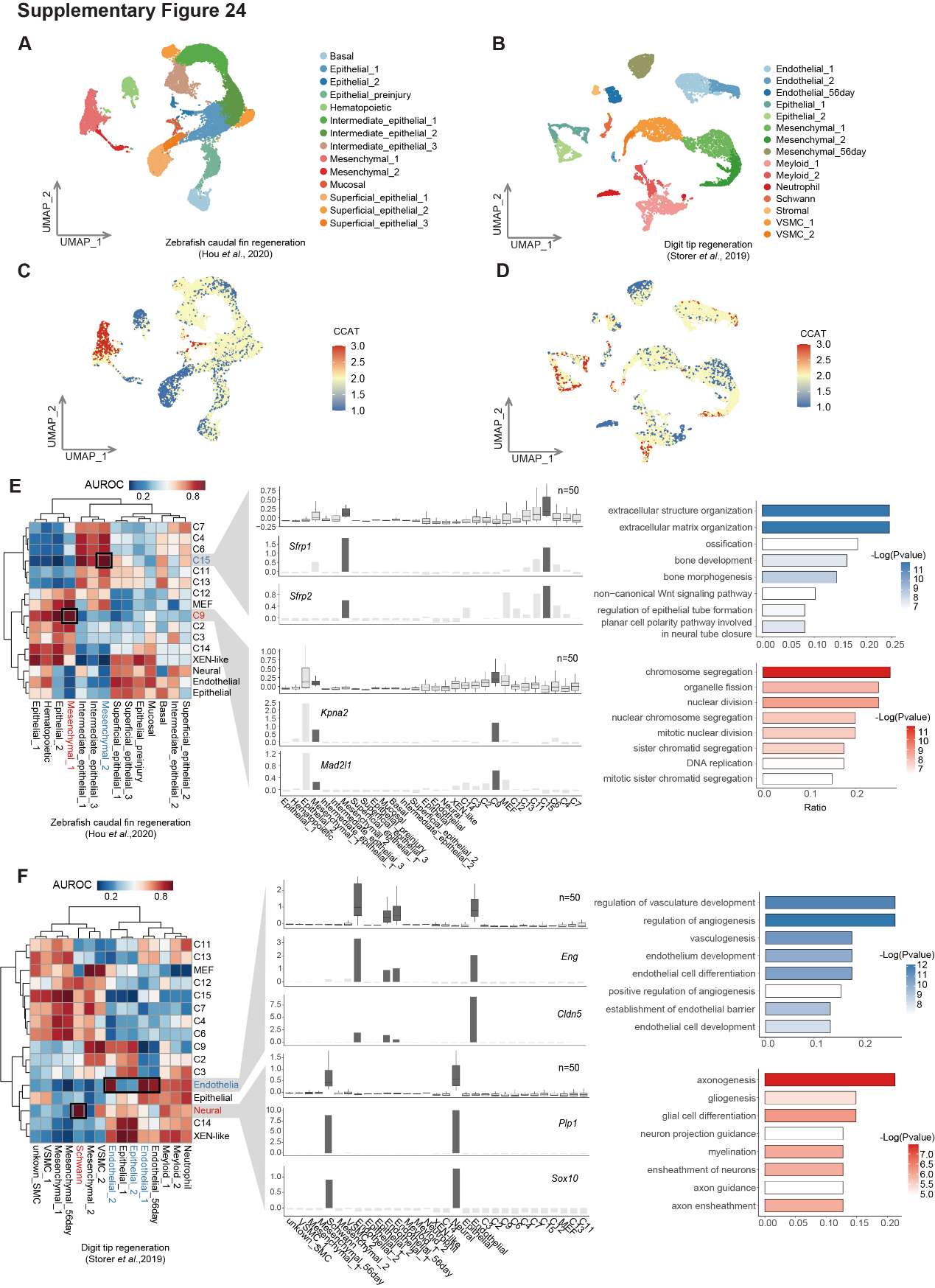


**Figure S24 Comparison between iMT and regeneration process.**

(A-B) UMAP visualizations of two regeneration data, zebrafish caudal fin regeneration (Hou *et al.*, 2020) (A) and mouse digit tip regeneration (Storer *et al.*, 2019) (B), colored by cell-type annotation.

(C-D) UMAP visualizations showing the distribution of CCAT entropy values of two regeneration data, zebrafish caudal fin regeneration (Hou *et al*., 2020) (C) and mouse digit tip regeneration (Storer *et al.*, 2019) (D), at single-cell level.

(E-F) The correlation analysis between cell types of iMT process (this work, column) and two regeneration data (row), zebrafish caudal fin regeneration (Hou *et al.*, 2020) (E) and mouse digit tip regeneration (Storer *et al.*, 2019) (F). Heatmaps showing the cell type correlation between cell types of iMT process (this work) and regeneration data (left). Boxplots showing the average expression of top driver genes promoting specific similarities between cell types in the two process (middle top). Barcharts showing the expression levels of two presentative genes (middle bottom). Representative GO terms enriched in the co-expressed driver genes (right).


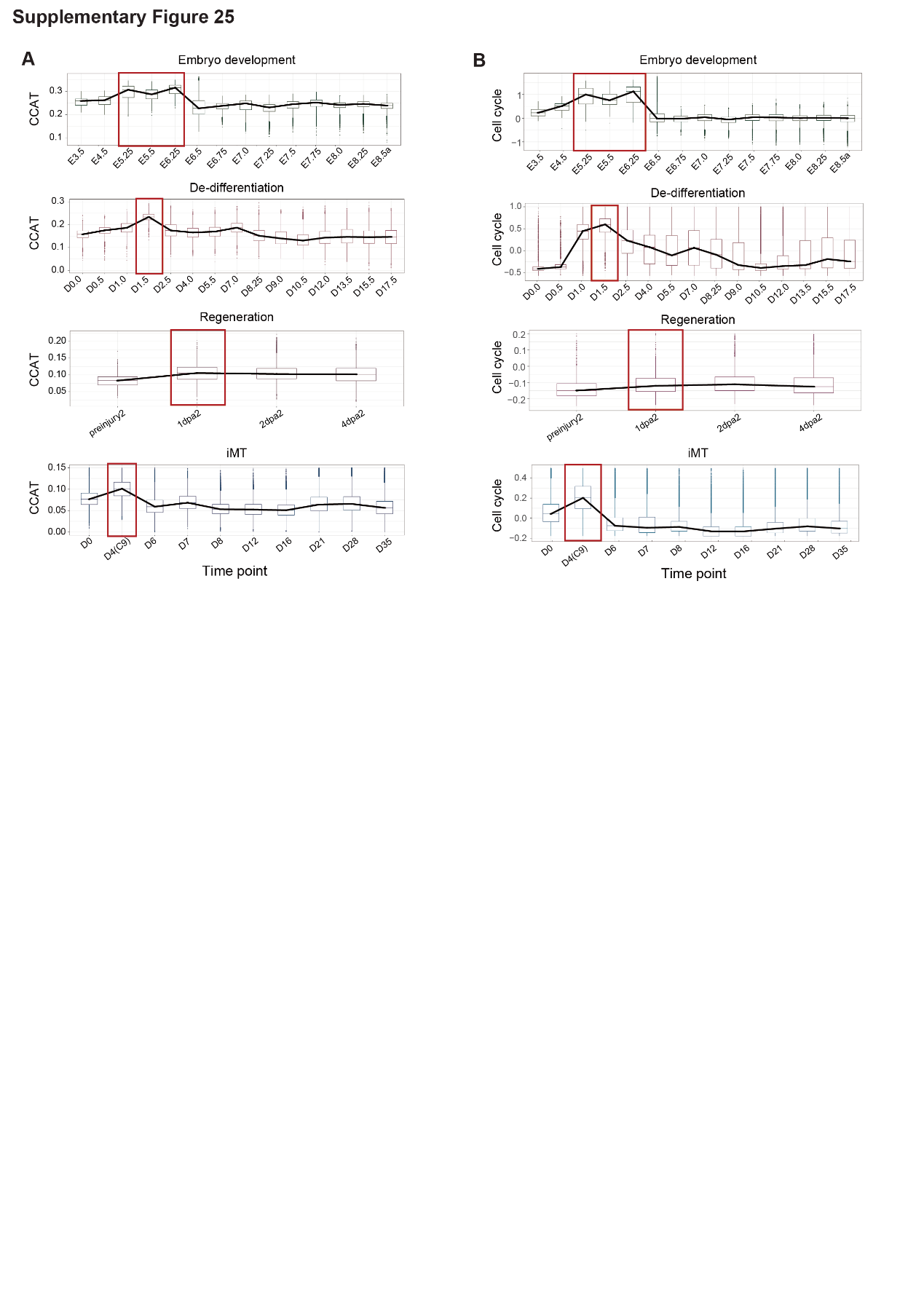


**Figure S25 A high entropy and cell-cycle score state during iMT, development and regeneration process.**

(A-B) Boxplots showing the distribution of the CCAT entropy values (A) and cell-cycle scores (B) across different time points in embryonic development (Qiu *et al.*, 2022), de-differentiation (Schiebinger *et al.*, 2019), regeneration (Hou *et al.*, 2020), and trans-differentiation (this work) from top to bottom.


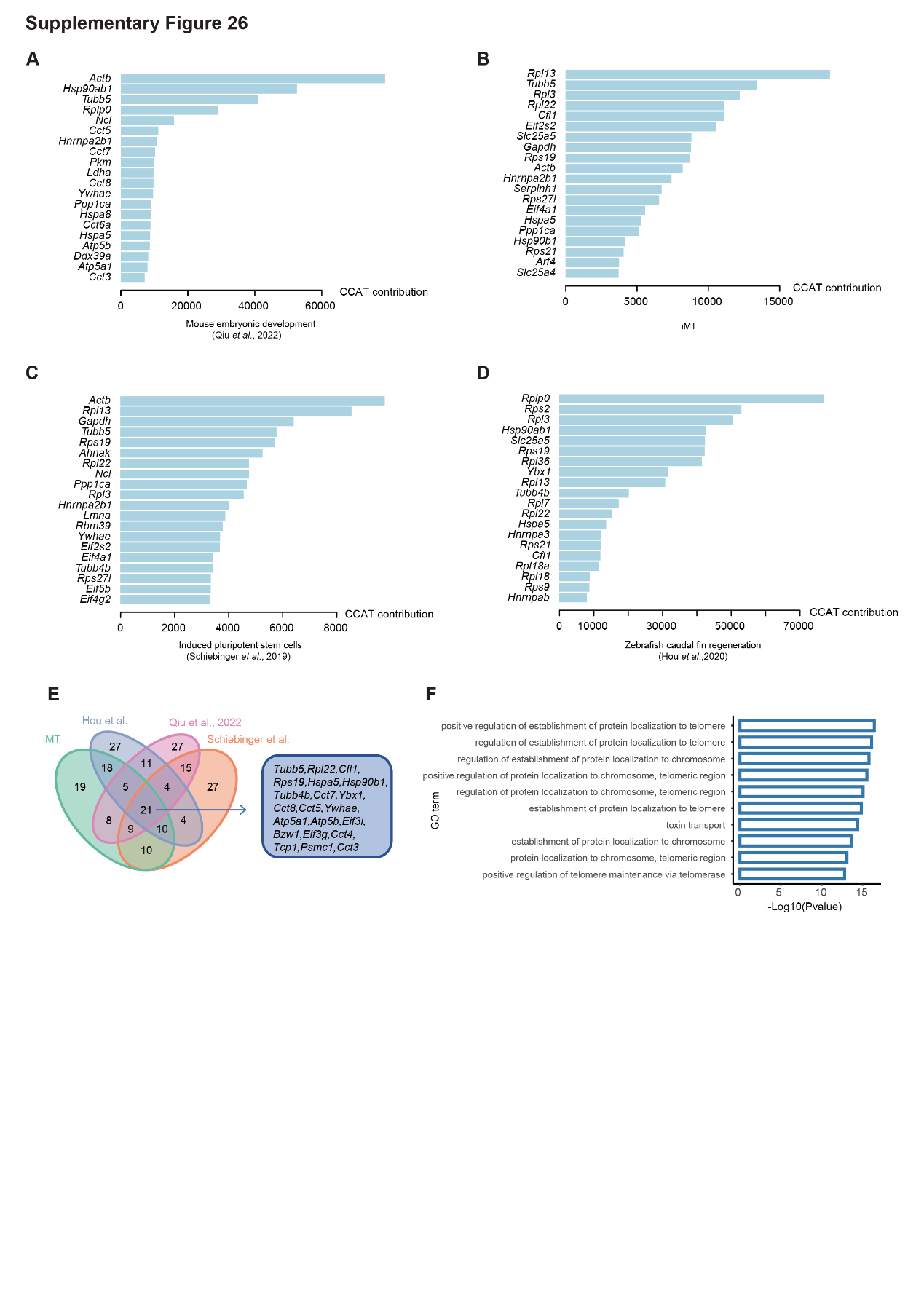


**Figure S26 Entropy values of high-entropy cells are mainly contributed by chromosome-related genes.**

(A-D) Barcharts showing the top 20 genes with CCAT contribution of high-entropy cells (cells highlight in Figure 6b) from mouse embryo development data (Qiu *et al.*, 2022) (A), iMT scRNA data (this work) (B), de-differentiation data (Schiebinger *et al.*, 2019) (C), and regeneration data (Hou *et al.*, 2020) (D).

(E) A venn diagram displaying the intersection of the top 100 genes contributing to entropy values across four datasets.

(F) A barchart showing the representative GO terms enriched in 21 intersection genes of the top 100 genes contributing to entropy values across four datasets.

**
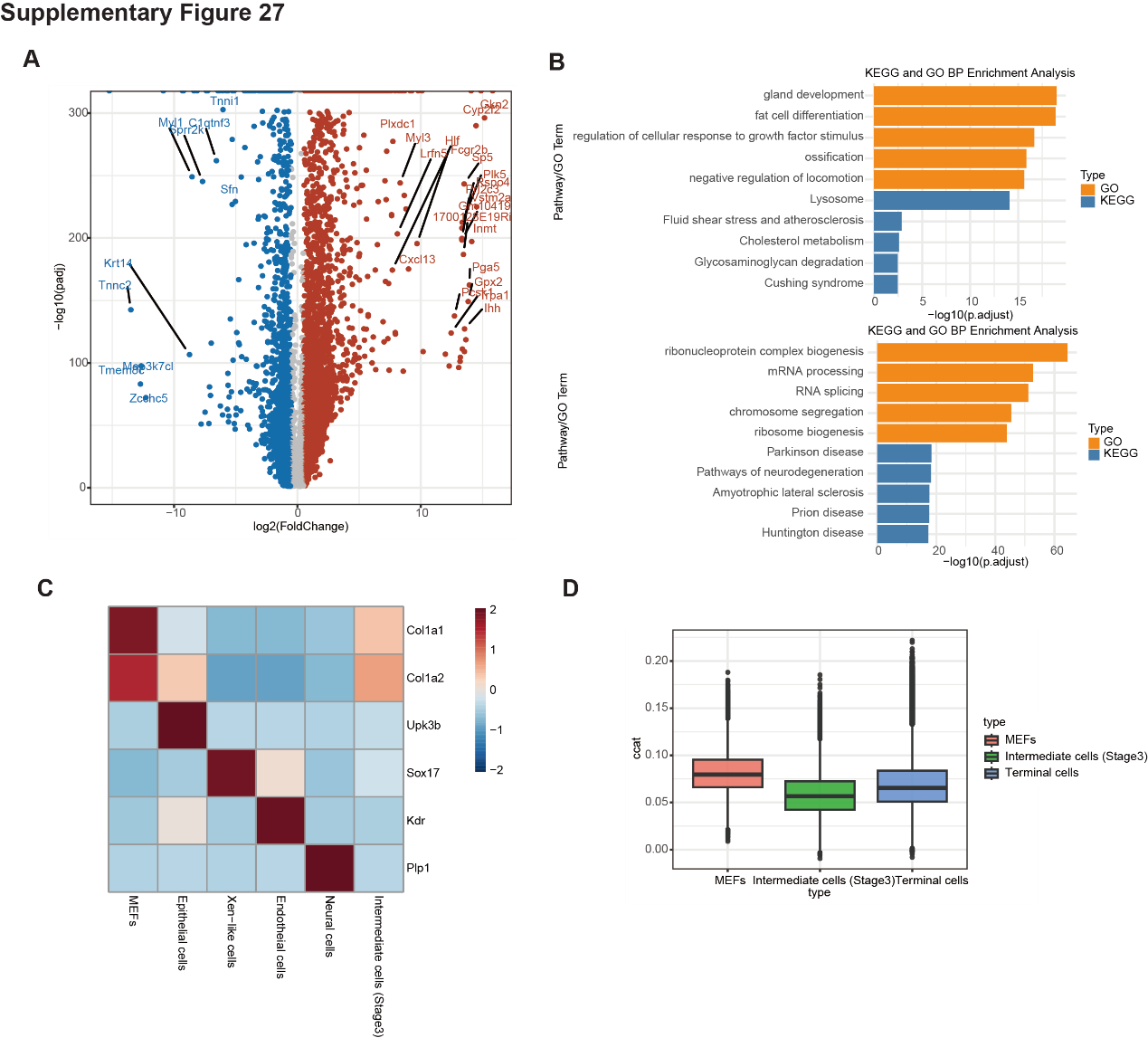
**

**Figure S27** **Differential gene expression and functional analysis across cell states.**

(A) A volcano plot showing the differential genes between MEFs and intermediate cells (stage3) from Microwell-seq data, where the upregulated genes in MEFs shown in red and the upregulated genes in intermediate cells (downregulated in MEFs) shown in blue.

(B) Barcharts shows the results of the GO BP and KEGG analysis for upregulated (top, stage3, upregulated in intermediate cells) and downregulated (bottom, upregulated in MEFs) genes, with the -log10(padj) of downregulated genes being negative.

(C) A heatmap demonstrating the gene expression of markers (columns) for different cell types (rows).

(D) A boxplot showing the distribution of the CCAT entropy values (columns) across different cell types (rows).
